# The LOVD3 platform: efficient genome-wide sharing of genetic variants

**DOI:** 10.1038/s41431-021-00959-x

**Published:** 2021-09-15

**Authors:** Ivo F.A.C. Fokkema, Mark Kroon, Julia A. López Hernández, Daan Asscheman, Ivar Lugtenburg, Jerry Hoogenboom, Johan T. den Dunnen

**Affiliations:** 1 Department of Human Genetics, Leiden University Medical Center, Leiden, Netherlands; 2 Department of Clinical Genetics, Leiden University Medical Center, Leiden, Netherlands

**Keywords:** Genetics research, Genetic predisposition to disease, Genetic databases

## Abstract

Gene variant databases are the backbone of DNA-based diagnostics. These databases, also called Locus-Specific DataBases (LSDBs), store information on variants in the human genome and the observed phenotypic consequences. The largest collection of public databases uses the free, open-source LOVD software platform. To cope with the current demand for online databases, we have entirely redesigned the LOVD software. LOVD3 is genome-centered and can be used to store summary variant data, as well as full case-level data with information on individuals, phenotypes, screenings, and variants. While built on a standard core, the software is highly flexible and allows personalization to cope with the largely different demands of gene/disease database curators. LOVD3 follows current standards and includes tools to check variant descriptions, generate HTML files of reference sequences, predict the consequences of exon deletions/duplications on the reading frame, and link to genomic views in the different genomes browsers. It includes APIs to collect and submit data. The software is used by about 100 databases, of which 56 public LOVD instances are registered on our website and together contain 1,000,000,000 variant observations in 1,500,000 individuals. 42 LOVD instances share data with the federated LOVD data network containing 3,000,000 unique variants in 23,000 genes. This network can be queried directly, quickly identifying LOVD instances containing relevant information on a searched variant.

## Introduction

Since next-generation sequencing, including whole-exome and whole-genome sequencing, has been introduced into the clinical practice, the classification of the variants identified has become the major bottleneck. Where classifying variants found in conventional single-gene screenings often used to be handled by an expert on the gene screened, specialists are now expected to classify variants for genes genome-wide. Even for a specific disease, the number of genes included in the disorder’s gene panel may go up to several hundred. The most helpful information to classify a variant is having direct access to all available data for the variant, including previous observations and the opinion from an expert in the field. To this end, expert-curated databases storing genetic variants and their classifications are an essential resource for any genome diagnostic lab.

For already more than 15 years, the open-source Leiden Open Variation Database (LOVD) software has enabled anybody to store and share gene variant data in a standardized database installation at their institute. LOVD2 (ref. [1].), with its first stable release in 2007, quickly became the most used software worldwide to establish locus-specific gene databases (LSDBs), with 99% of all LSDBs established using LOVD (January 2015). Its successor, the LOVD3 software, has been released in late 2012 and strengthened LOVD’s position as the most used LSDB software. Besides the option to install LOVD3 locally, thousands of users submit their data to the “Global Variome shared LOVD” instance, which also receives over half a million page views every month from tens of thousands of institutes. Besides diagnostics, LOVD is also heavily used in research. Google Scholar reports nearly 3000 citations or mentions of the software. Besides using LOVD to store or confirm findings in diagnostics, publications report using LOVD to study disease mechanisms such as the relationship between variant type and disease severity or the protein domain affected and the phenotype, to find targets for drug development, or to highlight variants common in specific populations.

There are several benefits LOVD has over other online genetic variant resources. Although LOVD does allow users to store only summary data for variants, in general, variants are presented with full case-level data; a patient or family, detailed phenotype information, information on the screening for variants, and the variant(s) found. Storing case-level data allows for allele-specific storage of variants (haplotypes). Therefore, it can show how a particular combination of variants, located in *cis* or *trans*, influences a patient’s phenotype. Variants are stored as their HGVS description, both on the genomic level and the transcript level. Classifications can be given on the transcript level, indicating which transcript is affected by the variant’s presence and which is not. Submitters and gene experts (Curators) classify variants separately. LOVD can be used directly in the clinical workflow—a patient record can be stored first, adding phenotype information only when it becomes available, followed by the screening, and finally, the variant data when the screening results come back. When needed, family relations can be stored to construct a pedigree.

LOVD is built to be flexible but still integrates with other LOVD instances. To accommodate the needs of different curators and to easily handle a myriad of disorders, LOVD uses the “custom column” feature to allow curators to set up which fields they want to use. As such, curators wishing to store detailed functional assay results can easily do so, whereas others can store detailed longitudinal phenotypic data of their cases, all in one LOVD instance. LOVD follows the HGVS recommendations for databases; data fields critical to guarantee minimal data quality can not be removed, such as the RNA field, which many other databases do not record. Using the RNA field, submitters can record confirmed cases of aberrant splicing, a popular and crucial feature. The “Global Variome shared LOVD” instance contains nearly 5000 records of confirmed RNA aberrant splicing as a result of single-base substitutions on the DNA level. The distributed nature of local LOVD instances supports the wish of some institutes to have full control over what data gets shared; specific fields or records can be hidden from the public when needed. Users can get viewing or editing rights to confidential records and fields in various ways, including gene-based authorization or specific authorization granted to data only from particular data owners. Local LOVD instances can be configured to be published in the list of instances on LOVD.nl, allowing their public variants to be included in the UCSC and Ensembl LOVD genome browser tracks and aiding their discovery using the LOVD search interfaces available worldwide.

### Introducing LOVD3

LOVD3 has been developed to meet several shortcomings of its LOVD2 predecessor. LOVD2’s popularity resulted in many different instances, focusing on different genes and disorders and having widely different custom column setups. LOVD3 makes phenotype columns disease-specific, and transcript variant columns are gene-specific, allowing these LOVD2 instances to be merged into one, greatly simplifying their maintenance. Also, upcoming NGS technologies shifted the focus from gene-based variants towards genomic variants. Therefore, LOVD3 allows for intergenic variants without mapping to any gene, as well as variants mapped to several genes, while maintaining the popular gene-based data views. NGS data can be imported from VCF files or submitted to LOVD from LIMS software using the LOVD submission API. Also, the data retrieval APIs were improved to promote using LOVD in variant annotation tools. Here, we highlight the most relevant new features of the LOVD3 software and introduce new ways LOVD can help classifying genetic variants.

## Materials and methods

### System requirements and installation

LOVD is designed to be cross-platform software, running on Linux, Unix, Windows, and macOS systems. It is written in the PHP programming language (https://php.net) and stores all data in a MySQL (https://mysql.com) or MariaDB (https://mariadb.org) database backend. As web-based software, LOVD requires running an HTTP web server, like Apache (https://httpd.apache.org). The combination of Apache, PHP, and MySQL or MariaDB is a widespread and popular choice. This makes it easy to find a server able to run LOVD or install the software required on a personal computer. Solutions such as XAMPP (https://apachefriends.org) install Apache, PHP, and MariaDB as one package on Linux, Windows, or Macintosh computers. The LOVD3 software itself is freely available from the LOVD website at https://LOVD.nl/download. The most recent development version can be found on GitHub at https://github.com/LOVDnl/LOVD3. GitHub is used to submit bug reports or feature requests and can be used to fork the repository. Documentation on the installation and use of LOVD can be found in the “docs” directory that comes with LOVD and online at https://lovd.nl/docs. The installation of LOVD generally takes only a few minutes. Once installed, LOVD will check for updates daily, providing information about the new update and a download link in case one is available. The Database Administrator or Manager is responsible for installing a LOVD update, which is as simple as overwriting the existing files with the new download and logging into LOVD.

### Data model changes

The LOVD3 data model is a complete redesign of the LOVD2 data model. The new data model was needed as LOVD2 merely annotated variants on the transcript level with a genomic position, while LOVD3 is ‘genome-centered’, storing genomic variants first and optionally linking these to variants on the transcript level. LOVD allows a flexible set of columns to be configured, complicating how data is stored. Different implementations of possible data models were tested and scored for speed of data querying, data insertion, custom column changes, and the required complexity of database queries and required storage space.

#### Custom column feature

LOVD’s custom column feature allows Curators and Managers to select in a gene-specific and disease-specific manner which columns are active for variants, screenings, individuals, and phenotypes. Relational database engines like MySQL and MariaDB need to rebuild a data table when columns are added or removed. Large tables take much time to be rebuilt; adding a column to a table that stores a million entries can take minutes to complete. An alternative approach is the “Entity Attribute Value” (EAV) model. Each data field for each entry is stored in a separate row, and no changes to the data model are required when fields should be added or removed. Empty fields do not necessarily need to be stored, saving disk space. As a downside, the EAV approach does require more complicated methods to insert, update, and retrieve data, potentially slowing down the use of the data table.

To determine which method would be most suitable for LOVD3, we implemented both approaches, storing 500.000 entries of data with each entry using only 25% of all data fields in the table. This sporadic use of active fields is typical in instances containing many genes or diseases, each with many different columns. The time required to store all entries and the resulting disk space usage was measured. Several searching methods were tested and timed while ensuring the result sets matched.

#### Variant tables

Using a ‘genome-centered’ setup allows storing variants not affecting a transcript (intergenic) and variants with annotation on multiple transcripts. However, it does require the use of at least two database tables in a one-to-many table relationship. We explored grouping variants in the database, storing unique variants only once as single entries. Data specific to the variant’s observation, like timestamps, users associated with the record, classifications, and references, would need to be stored elsewhere. Separating this information causes the variant information to be split over four tables. Although relational database design involves normalizing the database structure to reduce data duplication, joining four tables in one query when retrieving variant records is not feasible. We performed speed tests to measure the impact of full normalization of variant data into four tables and noticed a significant delay. Also, different variants on the genome level can result in one unique variant on the transcript level. Finally, variants detected only on the RNA level have a different variant description than those detected on the DNA level. This leaves hardly any field genuinely constant for one unique variant. Therefore, we decided to store all variant data in just two tables, one for all genomic data (VariantsOnGenome), one for all transcript-based data (VariantsOnTranscripts). Each variant observation is stored separately, regardless of how many times the variant has been recorded before. LOVD keeps track of unique variants using the DBID column in the VariantsOnGenome table.

#### Patient and phenotype data

LOVD2’s patient table has been renamed to “Individuals” to clarify the database can also be used to store data from healthy individuals, possibly with phenotypes that are not a disease (e.g., eye color, blood group, and bitter-tasting). LOVD2 stored phenotype data in the same table as the patient’s data, which caused problems with LOVD2 instances managing many different disorders. In such instances, the data entry form for creating a new patient record became very long and complicated, having data fields for an abundance of disorders. Also, this setup did not allow for the storage of longitudinal data. Splitting the phenotype data into a separate table in LOVD3 overcame these limitations, allowing custom column flexibility per disorder, phenotype records for several disorders separately attached to the same individual, and storing longitudinal phenotype data.

### Data integrity

To ensure the correct use of gene symbols, LOVD3 verifies the given gene symbols with the HGNC when new genes are created in LOVD3. The HGNC ID is also stored to ensure LOVD can track genes with updated symbols, and the HGNC ID can also be used to navigate to a gene. When creating transcript entries, external tools are used to verify the given transcript belongs to the given gene and can be used to map variants from and to the genome. For diseases, OMIM IDs are recommended, which will uniquely identify the disease and create a link to OMIM. Currently, OMIM does not have a registration-free API that allows us to verify disease names and symbols. However, a future version of LOVD3 will allow a Manager to input an OMIM API key such that these verifications will become possible.

### Database security

Web-based software is accessible by anyone with an internet connection, attracting many mostly automated attempts to break into the system or inject malware. LOVD has been online for more than 15 years and has been battle-tested against all known types of exploits. Examples are SQL injection, cross-site scripting (XSS) attacks, cross-site request forgery (CSRF), remote and local file inclusion attacks, session hijacking and fixation, and brute-forcing passwords. When accessing LOVD3 using the secure socket layer (SSL) protocol (using https:// rather than http:// in the address bar), all traffic between the user and LOVD is encrypted. This encryption prevents sensitive data or passwords from being read from the network. Users even have the option to restrict access to their accounts from only a specific IP address or IP address range, preventing access to their account even when others would have acquired the account’s password. Finally, when editing or removing data, the user is required to provide their password, preventing data manipulation from a computer left unattended after logging into LOVD.

### Access levels

Within LOVD, data entries or specific data fields can be hidden from public view. Non-public entries are accessible only to a select group of users. See Supplementary Table 1 for a tabular overview of all LOVD user levels. All LOVD users are data submitters, but additional rights and roles may be granted. Two global roles exist; the Database Administrator and the Manager. Roles assigned per gene are the Curator and the Collaborator. Finally, submitters can assign others as Colleagues. The Database Administrator and Managers are caretakers of the LOVD instance, who install and update LOVD, create genes and custom columns, assign rights to other users, and manage the LOVD’s settings. The Database Administrator and all Managers can view and edit all non-public data in the database. Curators, experts assigned in charge of handling data submissions to specific genes, can view and edit all public and non-public data only related to the gene(s) in their care. While the original classification from the submitter is always retained, the Curator may add their classification and optionally provide more background information to incoming submissions. Collaborators are Curators without edit rights, and they can view, but not edit, all public and non-public data related to the gene(s) to which they are assigned. Finally, any submitter can assign Colleagues to provide either view-only or full edit rights over the submitter’s data submissions, depending on their wishes. This makes data sharing between different users from one diagnostic laboratory or specific larger consortia easy to implement.

### Data APIs

#### Data retrieval API

LOVD2 introduced a gene-based data retrieval API, serving XML-based Atom feeds with plain-text payloads containing data on genes or variants. LOVD3 incorporated the same data retrieval API for backward compatibility, but with a few additions. Since LOVD3 allows for adding more than one transcript per gene, the LOVD3 data retrieval API, by default, selects the transcript with the most variants annotated. We added the additional JSON output format where selection is unnecessary, returning all of the gene’s variants. The JSON output format is much easier to parse since all popular programming languages have parsers available for this format. Also, it adds more fields compared to the LOVD2 XML-based API. It can share the variants’ genomic locations on multiple genomes builds if the LOVD instance in question stores these. Also, more metadata is shared like data owner, data creator, and the entry’s creation and last edit’s timestamps. However, using the LOVD2 structure for the data retrieval API does limit it to the retrieval of gene-based variants only, which will be solved with the introduction of a new data retrieval API in a later version of LOVD3.

#### Data submission API

LOVD3 features a data submission API that allows for automated submission of data from LIMS systems. The only manual steps required are registration of a submitter account at the LOVD instance and the subsequent creation of an API authentication token. The submission API is completely JSON-based, based on the open VarioML format [2]. The original VarioML format was XML-based, but it translates to JSON easily. The API checks the submission for missing mandatory fields or incorrect values and rapidly reports the outcome, after which the submission is queued for import into LOVD. The submission API is versioned, allowing backward-incompatible changes to be released without disturbing existing users’ workflows. Multiple versions can exist in parallel, giving users ample time to update their workflows if they choose to use a newer version.

#### Worldwide API

As LOVD is distributed software, it can be laborious to find information on a specific variant and check every LOVD instance. Therefore, we have created a worldwide API that returns results from all registered LOVD instances. It supports searching for genomic locations and genomic ranges and, optionally, a gene symbol. When a result is found, the API returns basic information, including a link to the variant record in the LOVD hosting the search result. LOVD3 instances use this API to find other records of any given variant using the “Search public LOVDs” feature. LOVDs can be registered to the LOVD website to be included in this feature by enabling the “Include in the global LOVD listing” option in the LOVD system settings.

The API has also been integrated into the optional LOVD module for the Ensembl VEP [3] software. A graphical interface for low-volume requests is available on the LOVD website, also showing the variant’s effect, if available.

### Upgrading from LOVD2

Because of the substantial changes in the data model, LOVD2 instances can not be upgraded to LOVD3 automatically, but the data have to be imported into an existing LOVD3 instance. First, the LOVD3 instance should have all the necessary genes and transcripts configured. Our “gene loader” script, available on https://github.com/LOVDnl/geneloader, can help create large numbers of genes and transcripts into LOVD3. Next, we have developed a script for LOVD2 that collects the necessary submitter accounts. The resulting export is a database script that checks if these accounts already exist in the target LOVD3 instance and otherwise creates them. Finally, LOVD2 gene database export files are converted by a LOVD2 data conversion script written for LOVD3 and subsequently imported into the system. We offer support to LOVD2 databases to move their data to a LOVD3 instance. We have merged thousands of genes from LOVD2 instances into the “Global Variome shared LOVD3” instance, bringing the data together all in one location.

## Results and discussion

General LOVD features are listed in Supplementary Table 2. The following sections will discuss LOVD3 features specifically.

### LOVD installation

The installation of LOVD3 is very straight-forward. First, the software can be downloaded from our website or GitHub and placed in a directory accessible for the webserver. Next, the configuration file should be copied and edited, providing information on the database backend. Subsequently, the system’s first user, the Database Administrator, fills in a simple web form with the account details. LOVD will install itself into the given database in less than a minute. After this, one more form is required to verify and complete the LOVD system settings. Most of these can also be changed at any later time, except for two specific settings; the uninstall lock and the reference sequence build. The uninstall lock disables the removal of LOVD3, and once enabled, it can only be removed by directly accessing the underlying database table. The reference sequence build defines the human genome build used by LOVD; hg19 or hg38. A future version of LOVD3 will allow configuring multiple genome builds. Other system settings added to LOVD3 include proxy settings for networks without a direct connection to the internet and the option to disable the registration form for new submitters. After installing the LOVD system, genes and transcripts must first be configured to annotate variants. When many genes and transcripts are required, the “gene loader” script can quickly create these. With downloads from the OMIM website, diseases can also be configured automatically using the “gene loader” script.

### Data model changes

**Fig. 1:**
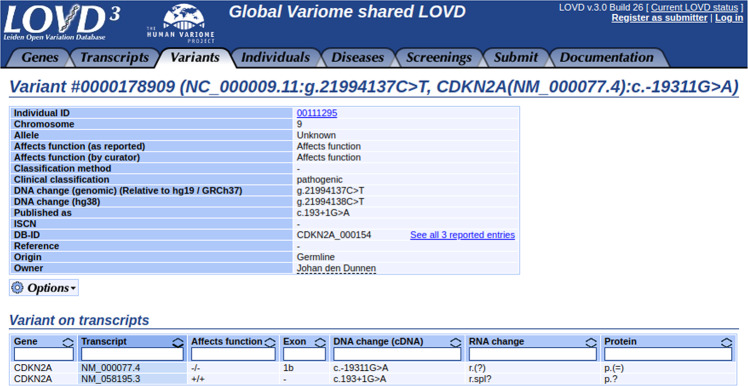
Any variant can be mapped to an unlimited number of transcripts, clearly displaying the variant’s effect on different isoforms. The variant’s effect can be set for each transcript separately.

The new data model allows storing intergenic variants and mapping variants to multiple genes and transcripts. Each gene can have multiple transcripts configured, for instance, when different isoforms are expressed that are relevant for different tissues or disease mechanisms. Variants can affect these transcripts differently, so the variant’s effect can be configured for all transcripts independently. This is a vital feature, which becomes evident in cases like the *CDKN2A* gene. *CDKN2A* produces multiple isoforms using two different reading frames that each has a unique function [4], notably NM_000077 and NM_058195. Clinical diagnostic laboratories may classify variants using only one of these transcripts. Therefore, classifications for variants reported by multiple laboratories may seem to conflict, while, in fact, they may have been classified using different transcripts. That this happens frequently is supported by the conflicting classifications found in the ClinVar [5] database for this gene. Of the 631 substitutions reported in ClinVar affecting *CDKN2A*, 62 (9.8%) are reported to have conflicting classifications (https://www.ncbi.nlm.nih.gov/clinvar/?term=CDKN2A%5Bgene%5D, visited 2020-07-17). We downloaded these 62 variants and used Variant Validator [6] to map them to the genome and then to both relevant transcripts, which was successful for 59 variants. Of these 59, 44 (74.6%) caused a missense or nonsense change on one of these two functional transcripts but a synonymous change on the other (see Supplementary Table 3). In some cases, evidence details confirmed that different transcripts were used to classify the variant (e.g., NC_000009.11:g.21971172C>T, https://www.ncbi.nlm.nih.gov/clinvar/variation/182417/, visited 2020-07-17). The percentage of this type of variant not reported to have conflicting classifications was only 52.6% (235 out of 447, *p* = 0.0013, Fisher’s exact test) (see Supplementary Table 4). To avoid confusion, the LOVD *CDKN2A* database at LOVD.nl/CDKN2A records variants’ effects on both transcripts (see Fig. 1). CDKN2A is not an isolated case. 178 genes in the same LOVD3 instance have configured more than one transcript.

Like in LOVD2, the variant effect is stored separately as given by the submitter and the curator. Next to the well-known gene-specific data views, LOVD3 introduces database-wide views for all genomic variants, per chromosome, or specific genomic regions only. Individuals and phenotypes can also be browsed and queried without selecting a gene first. The main views can be navigated using the menu tabs, while related entries always link to each other for navigation (see Fig. 1).

In LOVD3, each full submission (one patient and all their data) is stored in eight database tables (LOVD2 used three tables). The data submission process using the web forms has been redesigned into a step-by-step process, guiding the submitter through all necessary forms until the submission is complete. The submission wizard supports a clinical workflow, creating the individual and its phenotype first with detailed screening and variant data added later. LOVD3 supports longitudinal phenotype data, collecting phenotype data over time, following the disease or treatment progress (e.g., https://databases.lovd.nl/shared/individuals/00103738). LOVD3 allows indicating that a screening returned no results. Finally, any number of variants can be added. Data submitters must provide full submissions, including information on the individual as minimum quality assurance. However, Curators can submit variants without any individual attached, for instance, to summarize variant classification evidence. Users submitting data through the submission API can also be authorized to submit variant-only submissions if the remote system simply does not store any information on cases. This way, LOVD3 can be used for summary-level data, comparable to the ClinVar database, while still allowing the storage of complete cases when this data is available.

While a more sophisticated data model considerably enhances the database’s flexibility, it also complicates data exchange formats. One full database submission in LOVD2, taking data from three database tables, could still be represented in one spreadsheet table. This format made data imports relatively simple as often, data submitters already had large data sets prepared in spreadsheet tables. The eight database tables that LOVD3 requires for a full database submission can not be represented in a single spreadsheet table and need a more complex import format. The LOVD2 data conversion script (see “Materials and Methods” section) may be useful for submitters looking to convert their spreadsheet tables to the LOVD3 format.

#### Custom column feature optimization

To determine the most efficient method to implement LOVD’s custom column feature in LOVD3, we implemented both the traditional relational database table approach and the Entity Attribute Value (EAV) model. Surprisingly, the EAV method required more disk space in our setup (1109 MB versus 871 MB) even when not storing empty fields, likely due to the repetition of each data field’s primary key(s). This difference shows that traditional relational tables are quite optimized in handling empty fields. Inserting data was naturally much slower using the EAV approach, as many rows had to be created in the table for each entry represented. The EAV method completed the data insertion in 4430 s (74 min), while the traditional relational method needed only 513 s (8.6 min) to complete. Searching through both tables showed mixed results; when the search returned no results, the EAV table’s search finished 25% faster. When results were found, the relational table’s search completed between 11% and 56% faster, depending on data retrieval settings like other tables joined to, columns sorted on, and limitation of the number of results. Adding a column to the relational table took 110 s (1.8 min). Based on these results, we decided to use the traditional relational database table method for data storage. Changing the custom column configuration is relatively rare compared to data retrieval and data updates. The time penalty for custom column configuration changes is acceptable compared to the better storage size and (much) shorter insert and update times. When adding or removing a custom column and changing a data storage type, LOVD estimates the time it will take to rebuild the database table and warns the user accordingly.

Gene-specific or disease-specific custom columns can be enabled, disabled, and resorted by Curators. Some settings can also be configured for only specific genes or diseases, allowing Curators to easily configure the genes and the related diseases in their care without needing help from a Manager. LOVD3 comes with 43 pre-configured custom columns, but hundreds can be enabled simultaneously. The “Global Variome shared LOVD” instance currently has over 200 custom columns enabled, mostly phenotype columns. Having so many custom columns available makes genome-wide variant views and phenotype views challenging when not restricted to a single gene or disease. System-wide phenotype views in the “Global Variome shared LOVD” would need to show ~150 columns, of which in general only 8–10 are relevant for each entry. For this reason, currently, LOVD3 does not list phenotype records without selecting a disease first.

### Data APIs

**Fig. 2:**
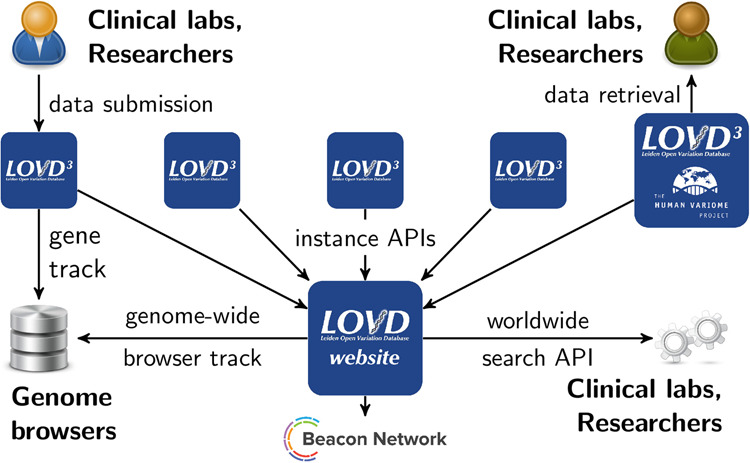
The full network of LOVD APIs. Clinical labs and researchers submit data to LOVD instances using data entry forms, upload files, or the submission API. LOVD instances can also be queried using their data retrieval API. The LOVD website aggregates variant data from registered LOVD instances worldwide and provides genome browser tracks (UCSC, Ensembl genome browsers), a link to the GA4GH Beacon Network (a global variant search engine), and the worldwide variant search API.

The network of LOVD APIs (see Fig. 2) consists of APIs present on each LOVD3 instance separately (data retrieval API and data submission API) and a central worldwide API hosted on LOVD.nl. Data from the worldwide API are shared with several third parties and enable LOVD genome-wide browser tracks in the UCSC and Ensembl genome browsers.

#### Data retrieval API

Over the last five years, usage of the data retrieval API made up 10–20% of all traffic to the “Global Variome shared LOVD” instance, highlighting the importance of non-human interaction with the databases. API traffic is likely to only increase with time, as (bio)informatics is becoming increasingly crucial for prioritizing variants in the diagnostic setting. The API allows listing all genes configured in the LOVD instance or searching for genes by gene symbol, chromosome, or chromosomal position. Variants can only be listed by gene, either showing all observations or grouping observations by unique variant description. Variants can be searched by position or range, either genomic or on the transcript, on DNA description, or the observation-independent database ID. The data retrieval API is also used to generate BED files, which are automatically loaded into the supported genome browsers when using the links from LOVD. This allows direct visualization of the variants in the UCSC and Ensembl genome browsers or the NCBI sequence viewer from any LOVD instance on a public network, even if this LOVD instance has not registered to be included in the worldwide LOVD network.

#### Data submission API

The data submission API was created on request from diagnostic labs seeking an automated solution to submit data directly to LOVD from their NGS LIMS systems. Submitters can create the necessary API token themselves, but new submissions will merely be scheduled for import and not directly imported by default. Submissions are imported as non-public entries, just like regular submissions done through the data entry forms. LOVD Managers can configure submissions to be automatically imported or even published as public entries on a per-user basis. This is useful for submissions from sources that already verify data intensively before submission and do not require verification by a LOVD Curator. The data submission API is a unique feature that is still missing in other database systems. Direct submission of data to LOVD is supported by several tools such as GSvar as part of ngs-bits (https://github.com/imgag/ngs-bits), MobiDetails [7] (https://mobidetails.iurc.montp.inserm.fr/MD/), and a soon to be released phenotype and variant curation and submission interface developed by the Medical Genetics Center Munich (MGZ).

#### Worldwide API

The worldwide API proves to be a valuable resource; in 2019, the API received more than 120 million requests, growing to 167 million requests in 2020. This number has already been doubled in the first few months of 2021. The API provides good coverage; 35.4% of requests provided at least one result (data from November 2020 through January 2021). The graphical interface on the LOVD website using the same API attracts a few thousand searches per month by several hundreds of unique IP addresses, summing up to several thousands of unique IP addresses a year. Also, LOVD itself has the option to search this API, allowing authorized users to check if a stored variant has been seen in any other LOVD instance worldwide. Having one interface to search all public LOVD instances dramatically reduces the time needed to find results, while at the same time allowing individual laboratories to easily share their results with the rest of the world by merely publishing their data in a public LOVD, possibly even hosted at their own institution. For instance, the Dutch genome diagnostic labs submit their data quarterly to LOVD to get their data included in the LOVD network [8], which has led to frequent matches and subsequent collaboration with international diagnostic laboratories (personal communication).

### Upgrading from LOVD2

At the peak of its usage, just before the release of LOVD3, some 100 LOVD2 public instances were registered to our public LOVD listing. LOVD3’s data model allows handling larger amounts of data and diverse diseases in one instance and helps reduce fragmentation of data. After years of migrating data to LOVD3 only on request, we have started to reduce the number of LOVD2 instances more actively. Currently, just 20 of the public LOVD instances remain. It should be noted that LOVD2 has reached end-of-life, and no further updates will be released. Anyone still hosting a LOVD2 instance is recommended to migrate their data to a LOVD3 instance or contact us for help. To simplify locating LOVD instances, we have established a series of short URLs. The LOVD.nl/<GENE> URL format (e.g., LOVD.nl/DMD) always redirects to the most up-to-date LOVD instance for a gene, preventing broken links when data were migrated from LOVD2 instances. More specifically, the URL LOVD.nl/DBID (e.g., LOVD.nl/DMD_000067) links to all entries of variant DMD_000067 (NM_004006.2:c.10141C>T) in the designated LOVD for this gene. The <GENE>.LOVD.nl URL format (e.g., DMD.LOVD.nl), automatically updated for public LOVD instances, shows all databases we know for a gene, including non-LOVD databases like ClinVar and Decipher [9].

### Other improvements

A standard LOVD instance links to other data sources to allow users to gather all information they need more quickly. From the gene home pages, links are provided to the genome browsers of the UCSC, Ensembl, and NCBI. Other links point to the HGNC, Entrez Gene, PubMed, OMIM, HGMD, GeneCards, and GeneTests. A statistics page is provided with various graphs showing statistics on this gene’s database contents. When LOVD is aware of the structure of the gene’s transcripts, a reading frame checker script is available to generate a prediction of the effect of whole-exon changes, being either frameshifting or not. Links to other resources can freely be added by the gene’s Curator. From the detailed variant view, links are provided to the UCSC and Ensembl genome browsers, automatically zooming into the variant’s location. When the variant is known to be found in the GnomAD dataset, LOVD also links to the GnomAD website. The link to the MobiDetails service will annotate the variant using a myriad of tools and present the results in a comprehensible overview. Finally, authorized users can look up the variant in the entire LOVD variant network using the “Search public LOVDs” feature.

To support editing data in bulk, LOVD3 offers Curators both a “find and replace” feature and an “update import” feature. For the latter, LOVD3 will detect the differences between the current data and an imported file and process only the changes detected. LOVD uses the data entries’ internal IDs to identify the entry in the database belonging to each line in the file. Only one change per entry is permitted to prevent accidentally replacing entries with others when mistyping their internal ID. When LOVD detects more than one change for any given entry, the entire file will be rejected. While performing extensive updates to the database or maintenance on the server hosting the LOVD instance, LOVD3 can be put into “read-only” mode, disallowing users to log into LOVD and showing a custom announcement that will be displayed on top of the screen.

**Fig. 3:**
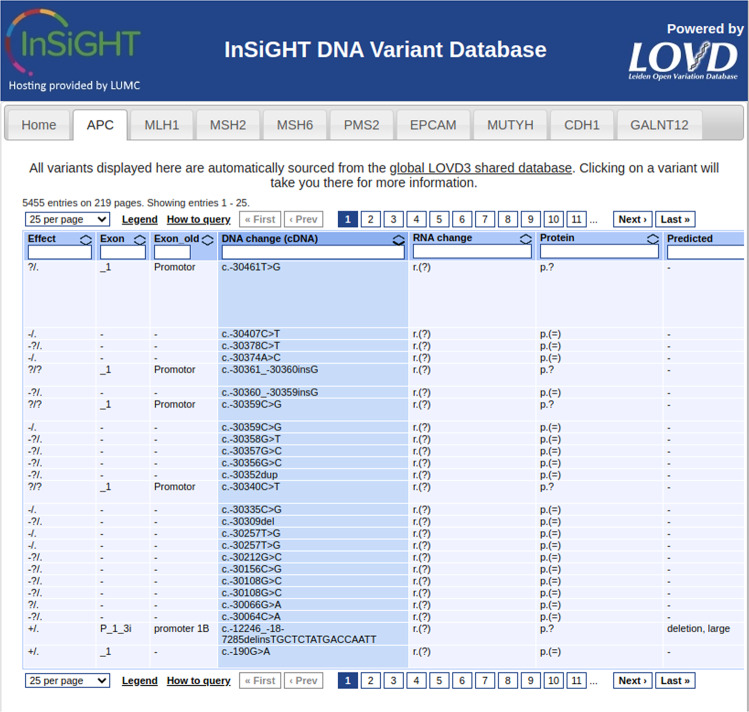
Example of the use of the “external LOVD data viewer” tool on the website of the database of the International Society for Gastrointestinal Hereditary Tumours (InSiGHT) (www.insight-database.org). The LOVD instance hosting this data provides only the data table below the gene tabs; the rest of the page can be designed however desired.

**Fig. 4:**
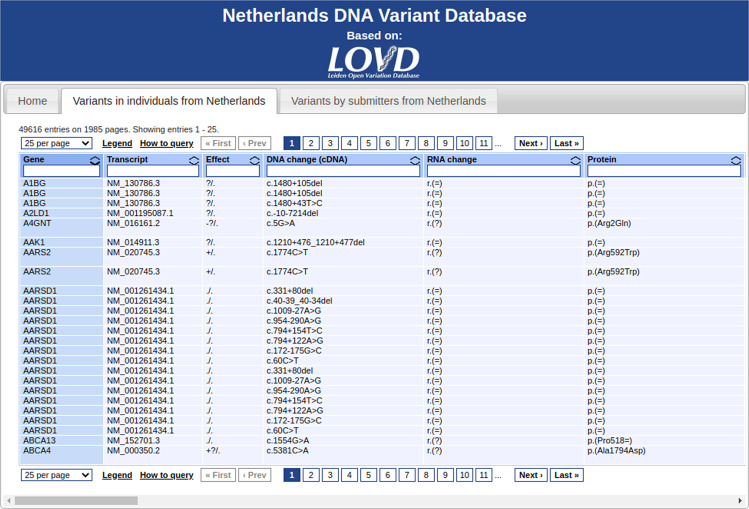
Example of using the “external LOVD data viewer” tool to create a country-specific data view (nl.lovd.org). The data shown on this page is retrieved real-time from the “Global Variome shared LOVD” instance.

To further help decrease database fragmentation, we created a tool (https://github.com/LOVDnl/external_view) that allows displaying data from any LOVD3 instance right on any PHP-enabled website. It can be used for submitters to showcase their submissions on their personal website or to show all data collected from patients only from a particular country. The displays are fully functional, allowing searching and sorting. Clicking on any entry will bring the user directly to the LOVD instance that provided the data. The variant database of the International Society for Gastrointestinal Hereditary Tumours (InSiGHT) [10] uses this method to display collected variants on their site (see Fig. 3). Another example of how this external data viewer tool can be implemented is the country node views accessible through <country>.lovd.org, e.g., nl.lovd.org or netherlands.lovd.org. These pages can be customized (e.g., mexico.lovd.org) and allow HVP country nodes to share their data through one central LOVD3 instance without creating separate databases. The pages show all Variants found in Individuals from that country and all Variants submitted by users from that country (see Fig. 4).

LOVD3 allows simple data retrieval based on the PubMedID or the publication’s DOI. When mentioned in the Reference field, URLs like https://databases.lovd.nl/shared/references/PMID:25533962 and https://databases.lovd.nl/shared/references/DOI:10.1038/nature14135 will retrieve all entries (Individuals and Variants) described in the publication. Authors/journals can use the link to refer to the database submission of their published data [11]. For DOIs, prefixes can also be used to refer to all data from this journal, e.g., https://databases.lovd.nl/shared/references/DOI:10.1038.

### Future developments

LOVD3 currently supports the use of only one genome build at a time; during installation, the genome build to use is selected, and from then on, all views, API calls, and submissions need to use this genome build. The “Global Variome shared LOVD” instance has been configured to use hg19/GRCh37 but added an hg38/GRCh38 DNA field, and we have included these mappings into the worldwide API. This, however, does not enable full support for hg38/GRCh38 API calls or submissions. Usage statistics of our worldwide API still show a nearly 50/50 balance of requests between these two genomes builds, showing users are still very divided. Adding full support for more than one genome build would allow LOVD to receive submissions and serve API requests using either build, serving the entire research and diagnostic community. We are currently working with the Variant Validator service (variantvalidator.org [6]) to provide fast and accurate mappings between builds to enable all variants in LOVD to be annotated on more than one build.

Although gene symbols are far more recognizable than the gene’s numeric HGNC IDs, gene symbols tend to change. We decided to use gene symbols in all views while storing the HGNC ID to uniquely identify each gene. We plan to develop a feature that will recognize changed gene symbols and notify the Curator. They can then choose to update the gene symbol throughout LOVD automatically. The replaced symbol will be stored as an alias to allow redirects and preserve links that users may have bookmarked.

The data retrieval API in LOVD3 still follows the LOVD2 style of gene-based queries and views. Also, very few fields are included in the output. We plan to develop a new JSON-based data retrieval API with many more fields included, allowing real-time genome-wide variant queries, phenotype-based queries, and retrieval of entire submissions, as well as aggregated data exports. When implemented, submitters will determine the data license for their submissions, deciding whether their data should be included at all, and if so, under which terms.

A future addition to the submission API will include being able to submit updates of previously submitted data. Currently, this requires logging into LOVD and submitting any changes through the data entry forms.

As LOVD3 is no longer strictly gene-based as LOVD2 was, the permission system has become significantly more complicated. Currently, Curators who only have access to the genes in their custody are not allowed to upload data files as these files can be used to upload public data in any gene or even outside of genes. Curators can simulate an import to verify that the imported file does not contain any errors. A manager should then verify the Curator’s rights regarding the data to be imported or edited and process the file. In a future release of LOVD3, LOVD will contain additional permission validation to enable Curators to process file imports.

### Example databases

We estimate there are currently some 100 LOVD instances worldwide. The 56 public LOVD instances published on our website (https://lovd.nl/public_list) together contain 1,000,000,000 variant observations in 1,500,000 individuals. The 42 instances included in the LOVD data network contain a total of 3,000,000 unique variants in 23,000 genes. Some of these instances are highlighted below.

The “Global Variome shared LOVD” (https://lovd.nl/shared) is the largest curated LOVD instance, hosting nearly 23,000 gene databases, collaboratively curated by 365 curators. Over 5000 new variant observations are submitted every month by over 2500 registered submitters. Data is submitted through the submission API, data entry web forms, or data file imports performed by the LOVD team. The Dutch genome diagnostic labs submit their data quarterly to this LOVD instance.

The Brazilian Initiative on Precision Medicine (BIPMed) [12] hosts several LOVD3 instances (https://bipmed.org/ourproducts), of which some are the largest known LOVD instances with nearly a million unique variants each.

The National Center for Scientific Research “Demokritos” from Greece hosts a LOVD3 instance cataloging germline variants in over 7000 Greek cancer patients, coined CanVaS (http://ithaka.rrp.demokritos.gr/CanVaS). It contains detailed phenotype information that is accessible upon request.

## Supplementary information

Supplementary Table 1

Supplementary Table 2

Supplementary Table 3

Supplementary Table 4

## Data Availability

All data generated or analysed during this study are included in this published article and its supplementary information files.
