# Supplementary material for: The LOVD3 platform: efficient genome-wide sharing of genetic variants
Source: Eur J Hum Genet. 2021 Sep 15;29(12):1796–803. doi: 10.1038/s41431-021-00959-x (PMC8632977; doi:10.1038/s41431-021-00959-x)
Supplement: Supplementary file 1 — Supplementary Table 1 [file 41431_2021_959_MOESM1_ESM.pdf]

Supplementary Table 1

Supplementary Table 1: Overview of LOVD3 user levels.

Level	Authorization based on	Description
**Submitter**		All LOVD users are submitters, but additional rights and roles can be granted.
**Colleague**	Other accounts	Submitters can assign 'colleague' rights (with or without edit rights) to other submitters. Colleagues are able to see all data from the other user, making data sharing between different users from one diagnostic laboratory or specific larger consortia easy to implement.
**Collaborator**	Gene	Is given read-only rights on a certain gene’s public and non-public data. Rights can be granted by a Manager or the Database Administrator.
**Curator**	Gene	Is given read/write rights on a certain gene’s public and non-public data. Receives emails about new data submissions relevant to the gene(s) they are assigned to. Can view and edit all public and non-public data related to the gene(s) in their care. Can configure transcripts and custom columns for the variant data related to their genes. Rights can be granted by a Manager or the Database Administrator.
**Manager**	Global	A caretaker of the LOVD instance, with the rights to update LOVD, create genes and custom columns, assign rights to other users, and manage the LOVD's settings. Can view and edit all non-public data in the database.
**Database Administrator**	Global	First user of the system, who installs LOVD. Can assign Managers to help taking care of the LOVD instance.
