# Supplementary material for: The LOVD3 platform: efficient genome-wide sharing of genetic variants
Source: Eur J Hum Genet. 2021 Sep 15;29(12):1796–803. doi: 10.1038/s41431-021-00959-x (PMC8632977; doi:10.1038/s41431-021-00959-x)
Supplement: Supplementary file 2 — Supplementary Table 2 [file 41431_2021_959_MOESM2_ESM.pdf]

Supplementary Table 2

Supplementary Table 2: General LOVD features, not specific to LOVD3.	
**General**	First released in 2004, LOVD is free, open-source software. LOVD was the first software package to receive the “recommended system” status from the Human Variome Project.<br>More than 95% of all gene variant databases worldwide are using LOVD, and LOVD has been cited or mentioned in thousands of scientific papers.
**Use cases**	The LOVD software can be used in many ways:<br>- Setting up a public or private local instance using LOVD, storing locally produced or collected data (bioinformaticians or clinicians/researchers with moderate computer skills)<br>- Setting up a patient registry using LOVD where patients own and control their own data (patient organisations)<br>- Setting up a central repository using LOVD for a collaboration between different institutes for high throughput data storage and data sharing using APIs and the graphical interface (clinicials/researchers supported by bioinformaticians)<br>- Using a single remote LOVD as an annotation source for diagnostic pipelines (bioinformaticians)<br>- Using the LOVD network as an annotation source for diagnostic pipelines (bioinformaticians)<br>- Using a remote LOVD instance for web-based data retrieval (clinicians/researchers)<br>- Setting up a local instance using LOVD to store a personal genome (anybody with moderate computer skills)
**Security**	LOVD contains built-in security measures against brute force attacks, SQL injection, cross-site scripting (XSS), cross-site request forgery (CSRF), local and remote file inclusion, and session hijacking and fixation.<br>Authorized user access can be limited to specific IP addresses, e.g., only from inside an institution. Detailed system log shows user activity and attempts to breach security measures.<br>Any data manipulation requires a password. Changes made through the data entry forms trigger an email to the data creator, owner, and Curator. LOVD uses a transactional database to protect against hardware failures.
**Flexible**	LOVD can run locally on personal computers or online on servers - Linux, Unix, Windows, and Mac are supported. LOVD allows personalization of the gene homepages and variant views allowing Curators to acknowledge funders and supporting institutes.<br>LOVD can store detailed case-level data or summary submissions only containing variant data. Cases can contain any number of variants, from a single variant result to millions of variants from a genome analysis.<br>Data can be added through data entry forms, file imports, or the submission API.<br>Detailed phenotype data can be stored on different diseases, including longitudinal data.<br>LOVD facilitates different ways to view and browse through the data. The custom column feature allows defining additional columns, including configuring the appearance on data entry forms.
**Integrated**	LOVD integrates tightly with Mutalyzer (mutalyzer.nl) and Variant Validator (variantvalidator.org), allowing to check the correct description of variants (according to HGVS standards) and to predict the effect of the variant on RNA and protein level. Variants can be automatically mapped from genomic positions to transcripts and vice-versa. LOVD links to the major genome browsers (UCSC, Ensembl, NCBI), displaying variants in their genomic context.<br>By enabling the "Include in the global LOVD listing" setting, the database name and location will be shown on the central LOVD website. Databases on this list are included in the LOVD querying services facilitating searches of LOVD instances containing a particular variant. Public variants will be included in the LOVD track in the major genome browsers. A full list of public databases (LOVD and others) for each gene is hosted on LOVD.nl/lsdbs.
**Powerful**	The data views allow for powerful search functionalities. Searching of records can be performed per column and allows the use of boolean search terms (AND, OR, and NOT). Curators can perform unique "find and replace" actions on all data or upload updated download files to quickly process large numbers of updates.
