# Supplementary material for: The LOVD3 platform: efficient genome-wide sharing of genetic variants
Source: Eur J Hum Genet. 2021 Sep 15;29(12):1796–803. doi: 10.1038/s41431-021-00959-x (PMC8632977; doi:10.1038/s41431-021-00959-x)
Supplement: Supplementary file 3 — Supplementary Table 3 [file 41431_2021_959_MOESM3_ESM.pdf]

Supplementary Table 3

Supplementary Table 3: The 59 substitutions affecting CDKN2A reported with conflicting classifications in ClinVar (https://www.ncbi.nlm.nih.gov/clinvar/?term=CDKN2A%5Bgene%5D, visited 2020-07-17) that we successfully mapped to both relevant transcripts using Variant Validator. Of these variants, 44 (74.6%) caused a missense or nonsense change on one of these two functional transcripts but a synonymous change on the other.

ClinVar variant description	Genomic description	NM_000077.4 protein description	NM_058195.3 protein description
NM_000077.4(CDKN2A):c.*8A>T	NC_000009.11:g.21968220T>A	p.(=)	p.(=)
NM_000077.4(CDKN2A):c.458-4G>C	NC_000009.11:g.21968245C>G	p.?	p.?
NM_000077.4(CDKN2A):c.402G>A	NC_000009.11:g.21970956C>T	p.(Ala134=)	p.(=)
NM_000077.4(CDKN2A):c.379G>C	NC_000009.11:g.21970979C>G	p.(Ala127Pro)	p.(=)
NM_000077.4(CDKN2A):c.373G>C	NC_000009.11:g.21970985C>G	p.(Asp125His)	p.(=)
NM_000077.4(CDKN2A):c.370C>T	NC_000009.11:g.21970988G>A	p.(Arg124Cys)	p.(=)
NM_000077.4(CDKN2A):c.369T>A	NC_000009.11:g.21970989A>T	p.(His123Gln)	p.(=)
NM_000077.4(CDKN2A):c.351G>C	NC_000009.11:g.21971007C>G	p.(Leu117=)	p.(Gly132Arg)
NM_000077.4(CDKN2A):c.342C>G	NC_000009.11:g.21971016G>C	p.(Pro114=)	p.(Arg129Gly)
NM_058195.3(CDKN2A):c.384C>T	NC_000009.11:g.21971017G>A	p.(Pro114Leu)	p.(Ala128=)
NM_000077.4(CDKN2A):c.339G>A	NC_000009.11:g.21971019C>T	p.(Leu113=)	p.(Ala128Thr)
NM_000077.4(CDKN2A):c.335G>T	NC_000009.11:g.21971023C>A	p.(Arg112Leu)	p.(Pro126=)
NM_000077.4(CDKN2A):c.322G>A	NC_000009.11:g.21971036C>T	p.(Asp108Asn)	p.(Arg122Gln)
NM_000077.4(CDKN2A):c.320G>A	NC_000009.11:g.21971038C>T	p.(Arg107His)	p.(Ala121=)
NM_000077.4(CDKN2A):c.318G>A	NC_000009.11:g.21971040C>T	p.(Val106=)	p.(Ala121Thr)
NM_058195.3(CDKN2A):c.358C>A	NC_000009.11:g.21971043G>T	p.(Asp105Glu)	p.(Arg120Ser)
NM_000077.4(CDKN2A):c.301G>T	NC_000009.11:g.21971057C>A	p.(Gly101Trp)	p.(Arg115Leu)
NM_000077.4(CDKN2A):c.300C>T	NC_000009.11:g.21971058G>A	p.(Ala100=)	p.(Arg115Trp)
NM_000077.4(CDKN2A):c.298G>T	NC_000009.11:g.21971060C>A	p.(Ala100Ser)	p.(Gly114Val)
NM_000077.4(CDKN2A):c.297G>T	NC_000009.11:g.21971061C>A	p.(Arg99=)	p.(Gly114Cys)
NM_000077.4(CDKN2A):c.294C>T	NC_000009.11:g.21971064G>A	p.(His98=)	p.(Pro113Ser)
NM_000077.4(CDKN2A):c.282G>A	NC_000009.11:g.21971076C>T	p.(Leu94=)	p.(Gly109Ser)
NM_000077.4(CDKN2A):c.273G>A	NC_000009.11:g.21971085C>T	p.(Leu91=)	p.(Gly106Arg)
NM_000077.4(CDKN2A):c.272T>A	NC_000009.11:g.21971086A>T	p.(Leu91Gln)	p.(Pro105=)
NM_000077.4(CDKN2A):c.266G>A	NC_000009.11:g.21971092C>T	p.(Gly89Asp)	p.(Gly103=)
NM_000077.4(CDKN2A):c.261G>A	NC_000009.11:g.21971097C>T	p.(Arg87=)	p.(Gly102Arg)
NM_000077.4(CDKN2A):c.251A>C	NC_000009.11:g.21971107T>G	p.(Asp84Ala)	p.(Arg98=)
NM_000077.4(CDKN2A):c.250G>T	NC_000009.11:g.21971108C>A	p.(Asp84Tyr)	p.(Arg98Leu)
NM_000077.4(CDKN2A):c.250G>A	NC_000009.11:g.21971108C>T	p.(Asp84Asn)	p.(Arg98Gln)
NM_000077.4(CDKN2A):c.249C>A	NC_000009.11:g.21971109G>T	p.(His83Gln)	p.(Arg98=)

NM_000077.4(CDKN2A):c.246G>A	NC_000009.11:g.21971112C>T	p.(Val82=)	p.(Ala97Thr)
NM_000077.4(CDKN2A):c.246G>C	NC_000009.11:g.21971112C>G	p.(Val82=)	p.(Ala97Pro)
NM_000077.4(CDKN2A):c.243C>T	NC_000009.11:g.21971115G>A	p.(Pro81=)	p.(Arg96Cys)
NM_000077.4(CDKN2A):c.242C>G	NC_000009.11:g.21971116G>C	p.(Pro81Arg)	p.(Thr95=)
NM_000077.4(CDKN2A):c.236C>T	NC_000009.11:g.21971122G>A	p.(Thr79Ile)	p.(His93=)
NM_000077.4(CDKN2A):c.225C>G	NC_000009.11:g.21971133G>C	p.(Pro75=)	p.(Arg90Gly)
NM_000077.4(CDKN2A):c.210C>T	NC_000009.11:g.21971148G>A	p.(Pro70=)	p.(Gln85Ter)
NM_000077.4(CDKN2A):c.206A>G	NC_000009.11:g.21971152T>C	p.(Glu69Gly)	p.(Gly83=)
NM_000077.4(CDKN2A):c.203C>T	NC_000009.11:g.21971155G>A	p.(Ala68Val)	p.(Arg82=)
NM_000077.4(CDKN2A):c.198C>T	NC_000009.11:g.21971160G>A	p.(His66=)	p.(Arg81Trp)
NM_000077.4(CDKN2A):c.197A>G	NC_000009.11:g.21971161T>C	p.(His66Arg)	p.(Pro80=)
NM_000077.4(CDKN2A):c.194T>C	NC_000009.11:g.21971164A>G	p.(Leu65Pro)	p.(Ala79=)
NM_000077.4(CDKN2A):c.186G>A	NC_000009.11:g.21971172C>T	p.(Leu62=)	p.(Ala77Thr)
NM_000077.4(CDKN2A):c.180G>A	NC_000009.11:g.21971178C>T	p.(Ala60=)	p.(Gly75Arg)
NM_000077.4(CDKN2A):c.174A>G	NC_000009.11:g.21971184T>C	p.(Arg58=)	p.(Ser73Gly)
NM_000077.4(CDKN2A):c.170C>T	NC_000009.11:g.21971188G>A	p.(Ala57Val)	p.(Arg71=)
NM_000077.4(CDKN2A):c.168C>T	NC_000009.11:g.21971190G>A	p.(Ser56=)	p.(Arg71Cys)
NM_000077.4(CDKN2A):c.151-4G>C	NC_000009.11:g.21971211C>G	p.?	p.?
NM_000077.4(CDKN2A):c.150+37G>C	NC_000009.11:g.21974640C>G	p.(=)	p.(=)
NM_000077.4(CDKN2A):c.150+12G>A	NC_000009.11:g.21974665C>T	p.(=)	p.(=)
NM_000077.4(CDKN2A):c.150+8G>A	NC_000009.11:g.21974669C>T	p.(=)	p.(=)
NM_000077.4(CDKN2A):c.149A>G	NC_000009.11:g.21974678T>C	p.(Gln50Arg)	p.(=)
NM_000077.4(CDKN2A):c.146T>C	NC_000009.11:g.21974681A>G	p.(Ile49Thr)	p.(=)
NM_000077.4(CDKN2A):c.104G>C	NC_000009.11:g.21974723C>G	p.(Gly35Ala)	p.(=)
NM_000077.4(CDKN2A):c.83T>G	NC_000009.11:g.21974744A>C	p.(Val28Gly)	p.(=)
NM_000077.4(CDKN2A):c.67G>C	NC_000009.11:g.21974760C>G	p.(Gly23Arg)	p.(=)
NM_000077.4(CDKN2A):c.67G>T	NC_000009.11:g.21974760C>A	p.(Gly23Cys)	p.(=)
NM_000077.4(CDKN2A):c.-14C>T	NC_000009.11:g.21974840G>A	p.(=)	p.(=)
NM_000077.4(CDKN2A):c.-34G>C	NC_000009.11:g.21974860C>G	p.(=)	p.(=)
