# Supplementary material for: The LOVD3 platform: efficient genome-wide sharing of genetic variants
Source: Eur J Hum Genet. 2021 Sep 15;29(12):1796–803. doi: 10.1038/s41431-021-00959-x (PMC8632977; doi:10.1038/s41431-021-00959-x)
Supplement: Supplementary file 4 — Supplementary Table 4 [file 41431_2021_959_MOESM4_ESM.pdf]

Supplementary Table 4

Supplementary Table 4: The 447 substitutions affecting CDKN2A not reported with conflicting classifications in ClinVar (https://www.ncbi.nlm.nih.gov/clinvar/?term=CDKN2A%5Bgene%5D, visited 2020-07-17) that we successfully mapped to both relevant transcripts using Variant Validator. Of these variants, 235 (52.6%) caused a missense or nonsense change on one of these two functional transcripts but a synonymous change on the other.

ClinVar variant description	Genomic description	NM_000077.4 protein description	NM_058195.3 protein description
NM_000077.4(CDKN2A):c.*13G>C	NC_000009.11:g.21968215C>G	p.(=)	p.(=)
NM_000077.4(CDKN2A):c.*9G>A	NC_000009.11:g.21968219C>T	p.(=)	p.(=)
NM_000077.4(CDKN2A):c.*9G>T	NC_000009.11:g.21968219C>A	p.(=)	p.(=)
NM_000077.4(CDKN2A):c.*8A>G	NC_000009.11:g.21968220T>C	p.(=)	p.(=)
NM_000077.4(CDKN2A):c.*6C>G	NC_000009.11:g.21968222G>C	p.(=)	p.(=)
NM_000077.4(CDKN2A):c.471A>C	NC_000009.11:g.21968228T>G	p.(*157Cysext*13)	p.(=)
NM_000077.4(CDKN2A):c.468T>C	NC_000009.11:g.21968231A>G	p.(Asp156=)	p.(=)
NM_000077.4(CDKN2A):c.466G>A	NC_000009.11:g.21968233C>T	p.(Asp156Asn)	p.(=)
NM_000077.4(CDKN2A):c.465C>G	NC_000009.11:g.21968234G>C	p.(Pro155=)	p.(=)
NM_000077.4(CDKN2A):c.465C>T	NC_000009.11:g.21968234G>A	p.(Pro155=)	p.(=)
NM_000077.4(CDKN2A):c.464C>G	NC_000009.11:g.21968235G>C	p.(Pro155Arg)	p.(=)
NM_000077.4(CDKN2A):c.463C>G	NC_000009.11:g.21968236G>C	p.(Pro155Ala)	p.(=)
NM_000077.4(CDKN2A):c.462C>A	NC_000009.11:g.21968237G>T	p.(Ile154=)	p.(=)
NM_000077.4(CDKN2A):c.461T>A	NC_000009.11:g.21968238A>T	p.(Ile154Asn)	p.(=)
NM_000077.4(CDKN2A):c.459C>T	NC_000009.11:g.21968240G>A	p.(Asp153=)	p.(=)
NM_000077.4(CDKN2A):c.458A>T	NC_000009.11:g.21968241T>A	p.(Asp153Val)	p.(=)
NM_000077.4(CDKN2A):c.458-2A>G	NC_000009.11:g.21968243T>C	p.?	p.?
NM_058195.3(CDKN2A):c.*102-9C>T	NC_000009.11:g.21968250G>A	p.(=)	p.(=)
NM_000077.4(CDKN2A):c.458-10C>G	NC_000009.11:g.21968251G>C	p.(=)	p.(=)
NM_058195.3(CDKN2A):c.*102-15T>A	NC_000009.11:g.21968256A>T	p.(=)	p.(=)
NM_058195.3(CDKN2A):c.*102-56G>T	NC_000009.11:g.21968297C>A	p.(=)	p.(=)
NM_058195.3(CDKN2A):c.*102-105A>G	NC_000009.11:g.21968346T>C	p.(=)	p.(=)
NM_058195.3(CDKN2A):c.*102-465G>C	NC_000009.11:g.21968706C>G	p.(=)	p.(=)
NM_058195.3(CDKN2A):c.*102-491C>T	NC_000009.11:g.21968732G>A	p.(=)	p.(=)
NM_058195.3(CDKN2A):c.*102-492G>C	NC_000009.11:g.21968733C>G	p.(=)	p.(=)
NM_000077.4(CDKN2A):c.458-525G>T	NC_000009.11:g.21968766C>A	p.(=)	p.(=)
NM_058195.3(CDKN2A):c.*102-541A>G	NC_000009.11:g.21968782T>C	p.(=)	p.(=)
NM_000077.4(CDKN2A):c.457+1129C>T	NC_000009.11:g.21969772G>A	p.(=)	p.(=)
NM_058195.3(CDKN2A):c.*101+929C>T	NC_000009.11:g.21969972G>A	p.(=)	p.(=)
NM_000077.4(CDKN2A):c.457+83C>T	NC_000009.11:g.21970818G>A	p.(=)	p.(=)

NM_000077.4(CDKN2A):c.457+30C>T	NC_000009.11:g.21970871G>A	p.(=)	p.(=)
NM_058195.3(CDKN2A):c.*101+15T>G	NC_000009.11:g.21970886A>C	p.(=)	p.(=)
NM_058195.3(CDKN2A):c.*101+12T>C	NC_000009.11:g.21970889A>G	p.(=)	p.(=)
NM_058195.3(CDKN2A):c.*101+11A>G	NC_000009.11:g.21970890T>C	p.(=)	p.(=)
NM_000077.4(CDKN2A):c.457+3G>A	NC_000009.11:g.21970898C>T	p.?	p.?
NM_000077.4(CDKN2A):c.457+2T>C	NC_000009.11:g.21970899A>G	p.?	p.?
NM_000077.4(CDKN2A):c.457+1G>A	NC_000009.11:g.21970900C>T	p.?	p.?
NM_000077.4(CDKN2A):c.457+1G>T	NC_000009.11:g.21970900C>A	p.?	p.?
NM_000077.4(CDKN2A):c.457G>A	NC_000009.11:g.21970901C>T	p.(Asp153Asn)	p.(=)
NM_000077.4(CDKN2A):c.457G>T	NC_000009.11:g.21970901C>A	p.(Asp153Tyr)	p.(=)
NM_000077.4(CDKN2A):c.455C>T	NC_000009.11:g.21970903G>A	p.(Ser152Leu)	p.(=)
NM_000077.4(CDKN2A):c.452C>T	NC_000009.11:g.21970906G>A	p.(Pro151Leu)	p.(=)
NM_000077.4(CDKN2A):c.450T>C	NC_000009.11:g.21970908A>G	p.(Gly150=)	p.(=)
NM_000077.4(CDKN2A):c.449G>A	NC_000009.11:g.21970909C>T	p.(Gly150Asp)	p.(=)
NM_000077.4(CDKN2A):c.447A>G	NC_000009.11:g.21970911T>C	p.(Glu149=)	p.(=)
NM_000077.4(CDKN2A):c.443C>T	NC_000009.11:g.21970915G>A	p.(Ala148Val)	p.(=)
NM_000077.4(CDKN2A):c.442G>C	NC_000009.11:g.21970916C>G	p.(Ala148Pro)	p.(=)
NM_000077.4(CDKN2A):c.442G>T	NC_000009.11:g.21970916C>A	p.(Ala148Ser)	p.(=)
NM_000077.4(CDKN2A):c.442G>A	NC_000009.11:g.21970916C>T	p.(Ala148Thr)	p.(=)
NM_000077.4(CDKN2A):c.441C>A	NC_000009.11:g.21970917G>T	p.(Ala147=)	p.(=)
NM_000077.4(CDKN2A):c.440C>T	NC_000009.11:g.21970918G>A	p.(Ala147Val)	p.(=)
NM_000077.4(CDKN2A):c.436G>C	NC_000009.11:g.21970922C>G	p.(Asp146His)	p.(=)
NM_000077.4(CDKN2A):c.436G>A	NC_000009.11:g.21970922C>T	p.(Asp146Asn)	p.(=)
NM_000077.4(CDKN2A):c.434T>A	NC_000009.11:g.21970924A>T	p.(Ile145Lys)	p.(=)
NM_000077.4(CDKN2A):c.434T>C	NC_000009.11:g.21970924A>G	p.(Ile145Thr)	p.(=)
NM_000077.4(CDKN2A):c.433A>G	NC_000009.11:g.21970925T>C	p.(Ile145Val)	p.(=)
NM_000077.4(CDKN2A):c.431G>C	NC_000009.11:g.21970927C>G	p.(Arg144Pro)	p.(=)
NM_000077.4(CDKN2A):c.431G>T	NC_000009.11:g.21970927C>A	p.(Arg144Leu)	p.(=)
NM_000077.4(CDKN2A):c.431G>A	NC_000009.11:g.21970927C>T	p.(Arg144His)	p.(=)
NM_000077.4(CDKN2A):c.430C>G	NC_000009.11:g.21970928G>C	p.(Arg144Gly)	p.(=)
NM_000077.4(CDKN2A):c.430C>T	NC_000009.11:g.21970928G>A	p.(Arg144Cys)	p.(=)
NM_000077.4(CDKN2A):c.429C>A	NC_000009.11:g.21970929G>T	p.(Ala143=)	p.(=)
NM_000077.4(CDKN2A):c.428C>G	NC_000009.11:g.21970930G>C	p.(Ala143Gly)	p.(=)
NM_000077.4(CDKN2A):c.427G>A	NC_000009.11:g.21970931C>T	p.(Ala143Thr)	p.(=)
NM_000077.4(CDKN2A):c.426T>G	NC_000009.11:g.21970932A>C	p.(His142Gln)	p.(=)
NM_000077.4(CDKN2A):c.425A>G	NC_000009.11:g.21970933T>C	p.(His142Arg)	p.(=)
NM_000077.4(CDKN2A):c.424C>G	NC_000009.11:g.21970934G>C	p.(His142Asp)	p.(=)

NM_000077.4(CDKN2A):c.424C>T	NC_000009.11:g.21970934G>A	p.(His142Tyr)	p.(=)
NM_000077.4(CDKN2A):c.421A>G	NC_000009.11:g.21970937T>C	p.(Asn141Asp)	p.(=)
NM_000077.4(CDKN2A):c.419G>A	NC_000009.11:g.21970939C>T	p.(Ser140Asn)	p.(=)
NM_000077.4(CDKN2A):c.418A>C	NC_000009.11:g.21970940T>G	p.(Ser140Arg)	p.(=)
NM_000077.4(CDKN2A):c.416G>A	NC_000009.11:g.21970942C>T	p.(Gly139Asp)	p.(=)
NM_000077.4(CDKN2A):c.415G>C	NC_000009.11:g.21970943C>G	p.(Gly139Arg)	p.(=)
NM_000077.4(CDKN2A):c.415G>A	NC_000009.11:g.21970943C>T	p.(Gly139Ser)	p.(=)
NM_000077.4(CDKN2A):c.412A>G	NC_000009.11:g.21970946T>C	p.(Arg138Gly)	p.(=)
NM_000077.4(CDKN2A):c.410C>T	NC_000009.11:g.21970948G>A	p.(Thr137Ile)	p.(=)
NM_000077.4(CDKN2A):c.407G>C	NC_000009.11:g.21970951C>G	p.(Gly136Ala)	p.(=)
NM_000077.4(CDKN2A):c.406G>A	NC_000009.11:g.21970952C>T	p.(Gly136Ser)	p.(=)
NM_000077.4(CDKN2A):c.405G>A	NC_000009.11:g.21970953C>T	p.(Gly135=)	p.(=)
NM_000077.4(CDKN2A):c.404G>T	NC_000009.11:g.21970954C>A	p.(Gly135Val)	p.(=)
NM_000077.4(CDKN2A):c.404G>A	NC_000009.11:g.21970954C>T	p.(Gly135Glu)	p.(=)
NM_000077.4(CDKN2A):c.402G>T	NC_000009.11:g.21970956C>A	p.(Ala134=)	p.(=)
NM_000077.4(CDKN2A):c.401C>T	NC_000009.11:g.21970957G>A	p.(Ala134Val)	p.(=)
NM_000077.4(CDKN2A):c.400G>T	NC_000009.11:g.21970958C>A	p.(Ala134Ser)	p.(=)
NM_000077.4(CDKN2A):c.400G>C	NC_000009.11:g.21970958C>G	p.(Ala134Pro)	p.(=)
NM_000077.4(CDKN2A):c.397G>A	NC_000009.11:g.21970961C>T	p.(Ala133Thr)	p.(=)
NM_000077.4(CDKN2A):c.396G>C	NC_000009.11:g.21970962C>G	p.(Ala132=)	p.(=)
NM_000077.4(CDKN2A):c.395C>T	NC_000009.11:g.21970963G>A	p.(Ala132Val)	p.(=)
NM_000077.4(CDKN2A):c.392G>A	NC_000009.11:g.21970966C>T	p.(Arg131His)	p.(=)
NM_000077.4(CDKN2A):c.388C>T	NC_000009.11:g.21970970G>A	p.(Leu130=)	p.(=)
NM_000077.4(CDKN2A):c.388C>A	NC_000009.11:g.21970970G>T	p.(Leu130Met)	p.(=)
NM_000077.4(CDKN2A):c.387C>T	NC_000009.11:g.21970971G>A	p.(Tyr129=)	p.(=)
NM_000077.4(CDKN2A):c.385T>G	NC_000009.11:g.21970973A>C	p.(Tyr129Asp)	p.(=)
NM_000077.4(CDKN2A):c.384G>A	NC_000009.11:g.21970974C>T	p.(Arg128=)	p.(=)
NM_000077.4(CDKN2A):c.383G>A	NC_000009.11:g.21970975C>T	p.(Arg128Gln)	p.(=)
NM_000077.4(CDKN2A):c.383G>C	NC_000009.11:g.21970975C>G	p.(Arg128Pro)	p.(=)
NM_000077.4(CDKN2A):c.382C>T	NC_000009.11:g.21970976G>A	p.(Arg128Trp)	p.(=)
NM_000077.4(CDKN2A):c.379G>T	NC_000009.11:g.21970979C>A	p.(Ala127Ser)	p.(=)
NM_000077.4(CDKN2A):c.377T>A	NC_000009.11:g.21970981A>T	p.(Val126Asp)	p.(=)
NM_000077.4(CDKN2A):c.376G>C	NC_000009.11:g.21970982C>G	p.(Val126Leu)	p.(=)
NM_000077.4(CDKN2A):c.374A>G	NC_000009.11:g.21970984T>C	p.(Asp125Gly)	p.(=)
NM_000077.4(CDKN2A):c.373G>A	NC_000009.11:g.21970985C>T	p.(Asp125Asn)	p.(=)
NM_000077.4(CDKN2A):c.371G>C	NC_000009.11:g.21970987C>G	p.(Arg124Pro)	p.(=)
NM_000077.4(CDKN2A):c.370C>A	NC_000009.11:g.21970988G>T	p.(Arg124Ser)	p.(=)

NM_000077.4(CDKN2A):c.370C>G	NC_000009.11:g.21970988G>C	p.(Arg124Gly)	p.(=)
NM_000077.4(CDKN2A):c.369T>C	NC_000009.11:g.21970989A>G	p.(His123=)	p.(=)
NM_000077.4(CDKN2A):c.365G>T	NC_000009.11:g.21970993C>A	p.(Gly122Val)	p.(=)
NM_058195.3(CDKN2A):c.*8G>C	NC_000009.11:g.21970994C>G	p.(Gly122Arg)	p.(=)
NM_000077.4(CDKN2A):c.362T>C	NC_000009.11:g.21970996A>G	p.(Leu121Pro)	p.(=)
NM_000077.4(CDKN2A):c.361C>T	NC_000009.11:g.21970997G>A	p.(Leu121=)	p.(=)
NM_000077.4(CDKN2A):c.361C>G	NC_000009.11:g.21970997G>C	p.(Leu121Val)	p.(=)
NM_000077.4(CDKN2A):c.360G>T	NC_000009.11:g.21970998C>A	p.(Glu120Asp)	p.(=)
NM_000077.4(CDKN2A):c.360G>A	NC_000009.11:g.21970998C>T	p.(Glu120=)	p.(=)
NM_000077.4(CDKN2A):c.355G>A	NC_000009.11:g.21971003C>T	p.(Glu119Lys)	p.(*133=)
NM_000077.4(CDKN2A):c.353C>T	NC_000009.11:g.21971005G>A	p.(Ala118Val)	p.(Gly132=)
NM_000077.4(CDKN2A):c.352G>A	NC_000009.11:g.21971006C>T	p.(Ala118Thr)	p.(Gly132Asp)
NM_000077.4(CDKN2A):c.351G>A	NC_000009.11:g.21971007C>T	p.(Leu117=)	p.(Gly132Ser)
NM_058195.3(CDKN2A):c.393T>C	NC_000009.11:g.21971008A>G	p.(Leu117Pro)	p.(Pro131=)
NM_000077.4(CDKN2A):c.349C>T	NC_000009.11:g.21971009G>A	p.(Leu117=)	p.(Pro131Leu)
NM_000077.4(CDKN2A):c.348C>A	NC_000009.11:g.21971010G>T	p.(Asp116Glu)	p.(Pro131Thr)
NM_000077.4(CDKN2A):c.344T>A	NC_000009.11:g.21971014A>T	p.(Val115Glu)	p.(Arg129=)
NM_000077.4(CDKN2A):c.344T>G	NC_000009.11:g.21971014A>C	p.(Val115Gly)	p.(Arg129=)
NM_000077.4(CDKN2A):c.343G>T	NC_000009.11:g.21971015C>A	p.(Val115Leu)	p.(Arg129Leu)
NM_058197.4(CDKN2A):c.*264C>A	NC_000009.11:g.21971017G>T	p.(Pro114His)	p.(Ala128=)
NM_058197.4(CDKN2A):c.*263C>A	NC_000009.11:g.21971018G>T	p.(Pro114Thr)	p.(Ala128Asp)
NM_058195.3(CDKN2A):c.381T>C	NC_000009.11:g.21971020A>G	p.(Leu113Pro)	p.(Ser127=)
NM_000077.4(CDKN2A):c.335G>A	NC_000009.11:g.21971023C>T	p.(Arg112His)	p.(Pro126=)
NM_000077.4(CDKN2A):c.334C>G	NC_000009.11:g.21971024G>C	p.(Arg112Gly)	p.(Pro126Arg)
NM_058197.4(CDKN2A):c.*256C>T	NC_000009.11:g.21971025G>A	p.(Gly111=)	p.(Pro126Ser)
NM_000077.4(CDKN2A):c.331G>A	NC_000009.11:g.21971027C>T	p.(Gly111Ser)	p.(Gly125Glu)
NM_000077.4(CDKN2A):c.331G>C	NC_000009.11:g.21971027C>G	p.(Gly111Arg)	p.(Gly125Ala)
NM_058197.4(CDKN2A):c.*253G>A	NC_000009.11:g.21971028C>T	p.(Trp110*)	p.(Gly125Arg)
NM_000077.4(CDKN2A):c.329G>A	NC_000009.11:g.21971029C>T	p.(Trp110*)	p.(Leu124=)
NM_000077.4(CDKN2A):c.327C>G	NC_000009.11:g.21971031G>C	p.(Ala109=)	p.(Leu124Val)
NM_000077.4(CDKN2A):c.326C>A	NC_000009.11:g.21971032G>T	p.(Ala109Asp)	p.(Cys123*)
NM_000077.4(CDKN2A):c.325G>T	NC_000009.11:g.21971033C>A	p.(Ala109Ser)	p.(Cys123Phe)
NM_000077.4(CDKN2A):c.325G>C	NC_000009.11:g.21971033C>G	p.(Ala109Pro)	p.(Cys123Ser)
NM_000077.4(CDKN2A):c.322G>T	NC_000009.11:g.21971036C>A	p.(Asp108Tyr)	p.(Arg122Leu)
NM_000077.4(CDKN2A):c.321C>G	NC_000009.11:g.21971037G>C	p.(Arg107=)	p.(Arg122Gly)
NM_000077.4(CDKN2A):c.320G>C	NC_000009.11:g.21971038C>G	p.(Arg107Pro)	p.(Ala121=)
NM_000077.4(CDKN2A):c.319C>T	NC_000009.11:g.21971039G>A	p.(Arg107Cys)	p.(Ala121Val)

NM_000077.4(CDKN2A):c.319C>A	NC_000009.11:g.21971039G>T	p.(Arg107Ser)	p.(Ala121Glu)
NM_000077.4(CDKN2A):c.317T>A	NC_000009.11:g.21971041A>T	p.(Val106Glu)	p.(Arg120=)
NM_000077.4(CDKN2A):c.317T>C	NC_000009.11:g.21971041A>G	p.(Val106Ala)	p.(Arg120=)
NM_058195.3(CDKN2A):c.358C>T	NC_000009.11:g.21971043G>A	p.(Asp105=)	p.(Arg120Cys)
NM_000077.4(CDKN2A):c.313G>T	NC_000009.11:g.21971045C>A	p.(Asp105Tyr)	p.(Gly119Val)
NM_000077.4(CDKN2A):c.313G>A	NC_000009.11:g.21971045C>T	p.(Asp105Asn)	p.(Gly119Glu)
NM_000077.4(CDKN2A):c.309G>A	NC_000009.11:g.21971049C>T	p.(Arg103=)	p.(Ala118Thr)
NM_000077.4(CDKN2A):c.308G>C	NC_000009.11:g.21971050C>G	p.(Arg103Pro)	p.(Ala117=)
NM_000077.4(CDKN2A):c.307C>T	NC_000009.11:g.21971051G>A	p.(Arg103Trp)	p.(Ala117Val)
NM_000077.4(CDKN2A):c.306G>T	NC_000009.11:g.21971052C>A	p.(Ala102=)	p.(Ala117Ser)
NM_000077.4(CDKN2A):c.305C>T	NC_000009.11:g.21971053G>A	p.(Ala102Val)	p.(Gly116=)
NM_000077.4(CDKN2A):c.304G>A	NC_000009.11:g.21971054C>T	p.(Ala102Thr)	p.(Gly116Asp)
NM_058195.3(CDKN2A):c.346G>T	NC_000009.11:g.21971055C>A	p.(Gly101=)	p.(Gly116Cys)
NM_000077.4(CDKN2A):c.302G>A	NC_000009.11:g.21971056C>T	p.(Gly101Glu)	p.(Arg115=)
NM_000077.4(CDKN2A):c.301G>A	NC_000009.11:g.21971057C>T	p.(Gly101Arg)	p.(Arg115Gln)
NM_000077.4(CDKN2A):c.301G>C	NC_000009.11:g.21971057C>G	p.(Gly101Arg)	p.(Arg115Pro)
NM_000077.4(CDKN2A):c.298G>A	NC_000009.11:g.21971060C>T	p.(Ala100Thr)	p.(Gly114Asp)
NM_000077.4(CDKN2A):c.297G>A	NC_000009.11:g.21971061C>T	p.(Arg99=)	p.(Gly114Ser)
NM_000077.4(CDKN2A):c.296G>A	NC_000009.11:g.21971062C>T	p.(Arg99Gln)	p.(Pro113=)
NM_000077.4(CDKN2A):c.296G>C	NC_000009.11:g.21971062C>G	p.(Arg99Pro)	p.(Pro113=)
NM_000077.4(CDKN2A):c.295C>T	NC_000009.11:g.21971063G>A	p.(Arg99Trp)	p.(Pro113Leu)
NM_000077.4(CDKN2A):c.295C>G	NC_000009.11:g.21971063G>C	p.(Arg99Gly)	p.(Pro113Arg)
NM_000077.4(CDKN2A):c.294C>G	NC_000009.11:g.21971064G>C	p.(His98Gln)	p.(Pro113Ala)
NM_000077.4(CDKN2A):c.292C>T	NC_000009.11:g.21971066G>A	p.(His98Tyr)	p.(Ala112Val)
NM_058195.3(CDKN2A):c.334G>C	NC_000009.11:g.21971067C>G	p.(Leu97=)	p.(Ala112Pro)
NM_000077.4(CDKN2A):c.288G>A	NC_000009.11:g.21971070C>T	p.(Val96=)	p.(Ala111Thr)
NM_000077.4(CDKN2A):c.286G>A	NC_000009.11:g.21971072C>T	p.(Val96Met)	p.(Gly110Asp)
NM_058195.3(CDKN2A):c.328G>A	NC_000009.11:g.21971073C>T	p.(Val95=)	p.(Gly110Ser)
NM_000077.4(CDKN2A):c.278C>G	NC_000009.11:g.21971080G>C	p.(Thr93Arg)	p.(His107Gln)
NM_000077.4(CDKN2A):c.276C>A	NC_000009.11:g.21971082G>T	p.(Asp92Glu)	p.(His107Asn)
NM_000077.4(CDKN2A):c.271C>T	NC_000009.11:g.21971087G>A	p.(Leu91=)	p.(Pro105Leu)
NM_000077.4(CDKN2A):c.269T>G	NC_000009.11:g.21971089A>C	p.(Phe90Cys)	p.(Leu104=)
NM_058195.3(CDKN2A):c.310C>T	NC_000009.11:g.21971091G>A	p.(Gly89=)	p.(Leu104Phe)
NM_000077.4(CDKN2A):c.266G>C	NC_000009.11:g.21971092C>G	p.(Gly89Ala)	p.(Gly103=)
NM_058197.4(CDKN2A):c.*188G>A	NC_000009.11:g.21971093C>T	p.(Gly89Ser)	p.(Gly103Glu)
NM_000077.4(CDKN2A):c.264G>A	NC_000009.11:g.21971094C>T	p.(Glu88=)	p.(Gly103Arg)
NM_000077.4(CDKN2A):c.262G>A	NC_000009.11:g.21971096C>T	p.(Glu88Lys)	p.(Gly102Glu)

NM_000077.4(CDKN2A):c.262G>C	NC_000009.11:g.21971096C>G	p.(Glu88Gln)	p.(Gly102Ala)
NM_000077.4(CDKN2A):c.262G>T	NC_000009.11:g.21971096C>A	p.(Glu88*)	p.(Gly102Val)
NM_000077.4(CDKN2A):c.260G>C	NC_000009.11:g.21971098C>G	p.(Arg87Pro)	p.(Pro101=)
NM_000077.4(CDKN2A):c.259C>T	NC_000009.11:g.21971099G>A	p.(Arg87Trp)	p.(Pro101Leu)
NM_000077.4(CDKN2A):c.258C>T	NC_000009.11:g.21971100G>A	p.(Ala86=)	p.(Pro101Ser)
NM_058195.3(CDKN2A):c.301C>A	NC_000009.11:g.21971100G>T	p.(Ala86=)	p.(Pro101Thr)
NM_000077.4(CDKN2A):c.253G>T	NC_000009.11:g.21971105C>A	p.(Ala85Ser)	p.(Arg99Leu)
NM_000077.4(CDKN2A):c.253G>A	NC_000009.11:g.21971105C>T	p.(Ala85Thr)	p.(Arg99His)
NM_058195.3(CDKN2A):c.295C>T	NC_000009.11:g.21971106G>A	p.(Asp84=)	p.(Arg99Cys)
NM_000077.4(CDKN2A):c.249C>T	NC_000009.11:g.21971109G>A	p.(His83=)	p.(Arg98*)
NM_058197.4(CDKN2A):c.*172C>G	NC_000009.11:g.21971109G>C	p.(His83Gln)	p.(Arg98Gly)
NM_058197.4(CDKN2A):c.*171A>G	NC_000009.11:g.21971110T>C	p.(His83Arg)	p.(Ala97=)
NM_058197.4(CDKN2A):c.*170C>G	NC_000009.11:g.21971111G>C	p.(His83Asp)	p.(Ala97Gly)
NM_000077.4(CDKN2A):c.247C>T	NC_000009.11:g.21971111G>A	p.(His83Tyr)	p.(Ala97Val)
NM_058195.3(CDKN2A):c.288T>A	NC_000009.11:g.21971113A>T	p.(Val82Glu)	p.(Arg96=)
NM_000077.4(CDKN2A):c.244G>C	NC_000009.11:g.21971114C>G	p.(Val82Leu)	p.(Arg96Pro)
NM_000077.4(CDKN2A):c.244G>T	NC_000009.11:g.21971114C>A	p.(Val82Leu)	p.(Arg96Leu)
NM_000077.4(CDKN2A):c.243C>G	NC_000009.11:g.21971115G>C	p.(Pro81=)	p.(Arg96Gly)
NM_058195.3(CDKN2A):c.285C>T	NC_000009.11:g.21971116G>A	p.(Pro81Leu)	p.(Thr95=)
NM_000077.4(CDKN2A):c.241C>T	NC_000009.11:g.21971117G>A	p.(Pro81Ser)	p.(Thr95Ile)
NM_000077.4(CDKN2A):c.241C>A	NC_000009.11:g.21971117G>T	p.(Pro81Thr)	p.(Thr95Asn)
NM_000077.4(CDKN2A):c.240A>G	NC_000009.11:g.21971118T>C	p.(Arg80=)	p.(Thr95Ala)
NM_000077.4(CDKN2A):c.239G>A	NC_000009.11:g.21971119C>T	p.(Arg80Gln)	p.(Pro94=)
NM_000077.4(CDKN2A):c.238C>G	NC_000009.11:g.21971120G>C	p.(Arg80Gly)	p.(Pro94Arg)
NM_000077.4(CDKN2A):c.238C>T	NC_000009.11:g.21971120G>A	p.(Arg80*)	p.(Pro94Leu)
NM_000077.4(CDKN2A):c.236C>A	NC_000009.11:g.21971122G>T	p.(Thr79Asn)	p.(His93Gln)
NM_000077.4(CDKN2A):c.235A>C	NC_000009.11:g.21971123T>G	p.(Thr79Pro)	p.(His93Pro)
NM_000077.4(CDKN2A):c.234C>T	NC_000009.11:g.21971124G>A	p.(Leu78=)	p.(His93Tyr)
NM_000077.4(CDKN2A):c.233T>C	NC_000009.11:g.21971125A>G	p.(Leu78Pro)	p.(Ser92=)
NM_000077.4(CDKN2A):c.232C>T	NC_000009.11:g.21971126G>A	p.(Leu78Phe)	p.(Ser92Phe)
NM_000077.4(CDKN2A):c.231T>C	NC_000009.11:g.21971127A>G	p.(Thr77=)	p.(Ser92Pro)
NM_000077.4(CDKN2A):c.230C>G	NC_000009.11:g.21971128G>C	p.(Thr77Ser)	p.(His91Gln)
NM_000077.4(CDKN2A):c.229A>G	NC_000009.11:g.21971129T>C	p.(Thr77Ala)	p.(His91Arg)
NM_000077.4(CDKN2A):c.226G>A	NC_000009.11:g.21971132C>T	p.(Ala76Thr)	p.(Arg90His)
NM_000077.4(CDKN2A):c.225C>T	NC_000009.11:g.21971133G>A	p.(Pro75=)	p.(Arg90Cys)
NM_000077.4(CDKN2A):c.225C>A	NC_000009.11:g.21971133G>T	p.(Pro75=)	p.(Arg90Ser)
NM_000077.4(CDKN2A):c.224C>T	NC_000009.11:g.21971134G>A	p.(Pro75Leu)	p.(Pro89=)

NM_000077.4(CDKN2A):c.223C>T	NC_000009.11:g.21971135G>A	p.(Pro75Ser)	p.(Pro89Leu)
NM_000077.4(CDKN2A):c.222C>G	NC_000009.11:g.21971136G>C	p.(Asp74Glu)	p.(Pro89Ala)
NM_000077.4(CDKN2A):c.221A>T	NC_000009.11:g.21971137T>A	p.(Asp74Val)	p.(Arg88=)
NM_058197.4(CDKN2A):c.*144A>C	NC_000009.11:g.21971137T>G	p.(Asp74Ala)	p.(Arg88=)
NM_058197.4(CDKN2A):c.*143G>C	NC_000009.11:g.21971138C>G	p.(Asp74His)	p.(Arg88Pro)
NM_000077.4(CDKN2A):c.220G>A	NC_000009.11:g.21971138C>T	p.(Asp74Asn)	p.(Arg88Gln)
NM_058195.3(CDKN2A):c.262C>A	NC_000009.11:g.21971139G>T	p.(Ala73=)	p.(Arg88=)
NM_000077.4(CDKN2A):c.219C>G	NC_000009.11:g.21971139G>C	p.(Ala73=)	p.(Arg88Gly)
NM_000077.4(CDKN2A):c.214T>A	NC_000009.11:g.21971144A>T	p.(Cys72Ser)	p.(Leu86Gln)
NM_000077.4(CDKN2A):c.212A>G	NC_000009.11:g.21971146T>C	p.(Asn71Ser)	p.(Gln85=)
NM_000077.4(CDKN2A):c.211A>T	NC_000009.11:g.21971147T>A	p.(Asn71Tyr)	p.(Gln85Leu)
NM_000077.4(CDKN2A):c.209C>T	NC_000009.11:g.21971149G>A	p.(Pro70Leu)	p.(Ala84=)
NM_000077.4(CDKN2A):c.209C>G	NC_000009.11:g.21971149G>C	p.(Pro70Arg)	p.(Ala84=)
NM_058195.3(CDKN2A):c.250G>A	NC_000009.11:g.21971151C>T	p.(Glu69=)	p.(Ala84Thr)
NM_058197.4(CDKN2A):c.*128G>T	NC_000009.11:g.21971153C>A	p.(Glu69*)	p.(Gly83Val)
NM_000077.4(CDKN2A):c.204G>A	NC_000009.11:g.21971154C>T	p.(Ala68=)	p.(Gly83Arg)
NM_000077.4(CDKN2A):c.203C>G	NC_000009.11:g.21971155G>C	p.(Ala68Gly)	p.(Arg82=)
NM_000077.4(CDKN2A):c.200G>A	NC_000009.11:g.21971158C>T	p.(Gly67Asp)	p.(Arg81=)
NM_000077.4(CDKN2A):c.199G>C	NC_000009.11:g.21971159C>G	p.(Gly67Arg)	p.(Arg81Pro)
NM_000077.4(CDKN2A):c.198C>G	NC_000009.11:g.21971160G>C	p.(His66Gln)	p.(Arg81Gly)
NM_000077.4(CDKN2A):c.198C>A	NC_000009.11:g.21971160G>T	p.(His66Gln)	p.(Arg81=)
NM_000077.4(CDKN2A):c.197A>C	NC_000009.11:g.21971161T>G	p.(His66Pro)	p.(Pro80=)
NM_000077.4(CDKN2A):c.195C>G	NC_000009.11:g.21971163G>C	p.(Leu65=)	p.(Pro80Ala)
NM_000077.4(CDKN2A):c.192G>T	NC_000009.11:g.21971166C>A	p.(Leu64=)	p.(Ala79Ser)
NM_058195.3(CDKN2A):c.229G>C	NC_000009.11:g.21971172C>G	p.(Leu62=)	p.(Ala77Pro)
NM_000077.4(CDKN2A):c.183G>C	NC_000009.11:g.21971175C>G	p.(Glu61Asp)	p.(Ala76Pro)
NM_058197.4(CDKN2A):c.*104G>T	NC_000009.11:g.21971177C>A	p.(Glu61*)	p.(Gly75Val)
NM_000077.4(CDKN2A):c.179C>T	NC_000009.11:g.21971179G>A	p.(Ala60Val)	p.(Gly74=)
NM_000077.4(CDKN2A):c.179C>A	NC_000009.11:g.21971179G>T	p.(Ala60Glu)	p.(Gly74=)
NM_000077.4(CDKN2A):c.178G>C	NC_000009.11:g.21971180C>G	p.(Ala60Pro)	p.(Gly74Ala)
NM_000077.4(CDKN2A):c.178G>A	NC_000009.11:g.21971180C>T	p.(Ala60Thr)	p.(Gly74Asp)
NM_058195.3(CDKN2A):c.220G>A	NC_000009.11:g.21971181C>T	p.(Val59=)	p.(Gly74Ser)
NM_000077.4(CDKN2A):c.176T>A	NC_000009.11:g.21971182A>T	p.(Val59Glu)	p.(Ser73Arg)
NM_000077.4(CDKN2A):c.176T>G	NC_000009.11:g.21971182A>C	p.(Val59Gly)	p.(Ser73Arg)
NM_000077.4(CDKN2A):c.174A>C	NC_000009.11:g.21971184T>G	p.(Arg58=)	p.(Ser73Arg)
NM_000077.4(CDKN2A):c.172C>G	NC_000009.11:g.21971186G>C	p.(Arg58Gly)	p.(Pro72Arg)
NM_000077.4(CDKN2A):c.172C>T	NC_000009.11:g.21971186G>A	p.(Arg58*)	p.(Pro72Leu)

NM_000077.4(CDKN2A):c.171C>T	NC_000009.11:g.21971187G>A	p.(Ala57=)	p.(Pro72Ser)
NM_058195.3(CDKN2A):c.213C>A	NC_000009.11:g.21971188G>T	p.(Ala57Asp)	p.(Arg71=)
NM_000077.4(CDKN2A):c.170C>G	NC_000009.11:g.21971188G>C	p.(Ala57Gly)	p.(Arg71=)
NM_000077.4(CDKN2A):c.168C>G	NC_000009.11:g.21971190G>C	p.(Ser56Arg)	p.(Arg71Gly)
NM_000077.4(CDKN2A):c.168C>A	NC_000009.11:g.21971190G>T	p.(Ser56Arg)	p.(Arg71Ser)
NM_000077.4(CDKN2A):c.167G>A	NC_000009.11:g.21971191C>T	p.(Ser56Asn)	p.(Gln70=)
NM_000077.4(CDKN2A):c.167G>T	NC_000009.11:g.21971191C>A	p.(Ser56Ile)	p.(Gln70His)
NM_058195.3(CDKN2A):c.207G>A	NC_000009.11:g.21971194C>T	p.(Gly55Asp)	p.(Gly69=)
NM_000077.4(CDKN2A):c.163G>C	NC_000009.11:g.21971195C>G	p.(Gly55Arg)	p.(Gly69Ala)
NM_000077.4(CDKN2A):c.162G>A	NC_000009.11:g.21971196C>T	p.(Met54Ile)	p.(Gly69Arg)
NM_000077.4(CDKN2A):c.161T>G	NC_000009.11:g.21971197A>C	p.(Met54Arg)	p.(Asp68Glu)
NM_000077.4(CDKN2A):c.161T>C	NC_000009.11:g.21971197A>G	p.(Met54Thr)	p.(Asp68=)
NM_058195.3(CDKN2A):c.203A>C	NC_000009.11:g.21971198T>G	p.(Met54Leu)	p.(Asp68Ala)
NM_000077.4(CDKN2A):c.159G>A	NC_000009.11:g.21971199C>T	p.(Met53Ile)	p.(Asp68Asn)
NM_000077.4(CDKN2A):c.159G>C	NC_000009.11:g.21971199C>G	p.(Met53Ile)	p.(Asp68His)
NM_000077.4(CDKN2A):c.156G>A	NC_000009.11:g.21971202C>T	p.(Met52Ile)	p.(Asp67Asn)
NM_000077.4(CDKN2A):c.155T>C	NC_000009.11:g.21971203A>G	p.(Met52Thr)	p.(His66=)
NM_000077.4(CDKN2A):c.151G>A	NC_000009.11:g.21971207C>T	p.(Val51Ile)	p.(Gly65Asp)
NM_000077.4(CDKN2A):c.151-1G>A	NC_000009.11:g.21971208C>T	p.?	p.?
NM_000077.4(CDKN2A):c.151-1G>T	NC_000009.11:g.21971208C>A	p.?	p.?
NM_000077.4(CDKN2A):c.151-1G>C	NC_000009.11:g.21971208C>G	p.?	p.?
NM_000077.4(CDKN2A):c.151-2A>C	NC_000009.11:g.21971209T>G	p.?	p.?
NM_000077.4(CDKN2A):c.151-2A>G	NC_000009.11:g.21971209T>C	p.?	p.?
NM_000077.4(CDKN2A):c.151-3C>A	NC_000009.11:g.21971210G>T	p.?	p.?
NM_000077.4(CDKN2A):c.151-7C>G	NC_000009.11:g.21971214G>C	p.(=)	p.(=)
NM_000077.4(CDKN2A):c.151-13T>C	NC_000009.11:g.21971220A>G	p.(=)	p.(=)
NM_000077.4(CDKN2A):c.151-14G>A	NC_000009.11:g.21971221C>T	p.(=)	p.(=)
NM_000077.4(CDKN2A):c.151-18T>C	NC_000009.11:g.21971225A>G	p.(=)	p.(=)
NM_000077.4(CDKN2A):c.151-66A>G	NC_000009.11:g.21971273T>C	p.(=)	p.(=)
NM_000077.4(CDKN2A):c.151-1238G>A	NC_000009.11:g.21972445C>T	p.(=)	p.(=)
NM_000077.4(CDKN2A):c.150+1255C>A	NC_000009.11:g.21973422G>T	p.(=)	p.(=)
NM_000077.4(CDKN2A):c.150+1104C>T	NC_000009.11:g.21973573G>A	p.(=)	p.(=)
NM_000077.4(CDKN2A):c.150+1104C>A	NC_000009.11:g.21973573G>T	p.(=)	p.(=)
NM_000077.4(CDKN2A):c.150+365T>G	NC_000009.11:g.21974312A>C	p.(=)	p.(=)
NM_000077.4(CDKN2A):c.150+280C>T	NC_000009.11:g.21974397G>A	p.(=)	p.(=)
NM_000077.4(CDKN2A):c.150+216A>G	NC_000009.11:g.21974461T>C	p.(=)	p.(=)
NM_000077.4(CDKN2A):c.150+193G>A	NC_000009.11:g.21974484C>T	p.(=)	p.(=)

NM_000077.4(CDKN2A):c.150+137T>G	NC_000009.11:g.21974540A>C	p.(=)	p.(=)
NM_000077.4(CDKN2A):c.150+82A>G	NC_000009.11:g.21974595T>C	p.(=)	p.(=)
NM_000077.4(CDKN2A):c.150+40C>T	NC_000009.11:g.21974637G>A	p.(=)	p.(=)
NM_000077.4(CDKN2A):c.150+30G>A	NC_000009.11:g.21974647C>T	p.(=)	p.(=)
NM_000077.4(CDKN2A):c.150+20C>G	NC_000009.11:g.21974657G>C	p.(=)	p.(=)
NM_000077.4(CDKN2A):c.150+20C>T	NC_000009.11:g.21974657G>A	p.(=)	p.(=)
NM_000077.4(CDKN2A):c.150+18A>G	NC_000009.11:g.21974659T>C	p.(=)	p.(=)
NM_000077.4(CDKN2A):c.150+11G>A	NC_000009.11:g.21974666C>T	p.(=)	p.(=)
NM_000077.4(CDKN2A):c.150+6T>C	NC_000009.11:g.21974671A>G	p.(=)	p.(=)
NM_000077.4(CDKN2A):c.150+5G>T	NC_000009.11:g.21974672C>A	p.?	p.(=)
NM_000077.4(CDKN2A):c.150+4G>A	NC_000009.11:g.21974673C>T	p.?	p.(=)
NM_000077.4(CDKN2A):c.150+2T>C	NC_000009.11:g.21974675A>G	p.?	p.(=)
NM_058195.3(CDKN2A):c.194-3470G>T	NC_000009.11:g.21974677C>A	p.(Gln50His)	p.(=)
NM_000077.4(CDKN2A):c.149A>C	NC_000009.11:g.21974678T>G	p.(Gln50Pro)	p.(=)
NM_058195.3(CDKN2A):c.194-3471A>T	NC_000009.11:g.21974678T>A	p.(Gln50Leu)	p.(=)
NM_058195.3(CDKN2A):c.194-3472C>A	NC_000009.11:g.21974679G>T	p.(Gln50Lys)	p.(=)
NM_000077.4(CDKN2A):c.148C>T	NC_000009.11:g.21974679G>A	p.(Gln50*)	p.(=)
NM_000077.4(CDKN2A):c.147C>G	NC_000009.11:g.21974680G>C	p.(Ile49Met)	p.(=)
NM_000077.4(CDKN2A):c.147C>A	NC_000009.11:g.21974680G>T	p.(Ile49=)	p.(=)
NM_000077.4(CDKN2A):c.146T>G	NC_000009.11:g.21974681A>C	p.(Ile49Ser)	p.(=)
NM_000077.4(CDKN2A):c.144G>A	NC_000009.11:g.21974683C>T	p.(Pro48=)	p.(=)
NM_000077.4(CDKN2A):c.143C>G	NC_000009.11:g.21974684G>C	p.(Pro48Arg)	p.(=)
NM_000077.4(CDKN2A):c.142C>A	NC_000009.11:g.21974685G>T	p.(Pro48Thr)	p.(=)
NM_000077.4(CDKN2A):c.137G>A	NC_000009.11:g.21974690C>T	p.(Arg46Gln)	p.(=)
NM_000077.4(CDKN2A):c.137G>C	NC_000009.11:g.21974690C>G	p.(Arg46Pro)	p.(=)
NM_000077.4(CDKN2A):c.136C>T	NC_000009.11:g.21974691G>A	p.(Arg46Trp)	p.(=)
NM_000077.4(CDKN2A):c.133G>C	NC_000009.11:g.21974694C>G	p.(Gly45Arg)	p.(=)
NM_000077.4(CDKN2A):c.133G>A	NC_000009.11:g.21974694C>T	p.(Gly45Ser)	p.(=)
NM_000077.4(CDKN2A):c.132C>A	NC_000009.11:g.21974695G>T	p.(Tyr44*)	p.(=)
NM_000077.4(CDKN2A):c.132C>T	NC_000009.11:g.21974695G>A	p.(Tyr44=)	p.(=)
NM_000077.4(CDKN2A):c.132C>G	NC_000009.11:g.21974695G>C	p.(Tyr44*)	p.(=)
NM_000077.4(CDKN2A):c.131A>G	NC_000009.11:g.21974696T>C	p.(Tyr44Cys)	p.(=)
NM_000077.4(CDKN2A):c.128G>C	NC_000009.11:g.21974699C>G	p.(Ser43Thr)	p.(=)
NM_000077.4(CDKN2A):c.127A>G	NC_000009.11:g.21974700T>C	p.(Ser43Gly)	p.(=)
NM_000077.4(CDKN2A):c.126T>G	NC_000009.11:g.21974701A>C	p.(Asn42Lys)	p.(=)
NM_000077.4(CDKN2A):c.125A>G	NC_000009.11:g.21974702T>C	p.(Asn42Ser)	p.(=)
NM_000077.4(CDKN2A):c.124A>G	NC_000009.11:g.21974703T>C	p.(Asn42Asp)	p.(=)

NM_000077.4(CDKN2A):c.122C>A	NC_000009.11:g.21974705G>T	p.(Pro41Gln)	p.(=)
NM_000077.4(CDKN2A):c.121C>G	NC_000009.11:g.21974706G>C	p.(Pro41Ala)	p.(=)
NM_000077.4(CDKN2A):c.118G>T	NC_000009.11:g.21974709C>A	p.(Ala40Ser)	p.(=)
NM_000077.4(CDKN2A):c.117C>G	NC_000009.11:g.21974710G>C	p.(Asn39Lys)	p.(=)
NM_000077.4(CDKN2A):c.117C>A	NC_000009.11:g.21974710G>T	p.(Asn39Lys)	p.(=)
NM_000077.4(CDKN2A):c.112C>T	NC_000009.11:g.21974715G>A	p.(Pro38Ser)	p.(=)
NM_000077.4(CDKN2A):c.111G>A	NC_000009.11:g.21974716C>T	p.(Leu37=)	p.(=)
NM_000077.4(CDKN2A):c.108G>C	NC_000009.11:g.21974719C>G	p.(Ala36=)	p.(=)
NM_000077.4(CDKN2A):c.107C>T	NC_000009.11:g.21974720G>A	p.(Ala36Val)	p.(=)
NM_000077.4(CDKN2A):c.106G>T	NC_000009.11:g.21974721C>A	p.(Ala36Ser)	p.(=)
NM_000077.4(CDKN2A):c.106G>A	NC_000009.11:g.21974721C>T	p.(Ala36Thr)	p.(=)
NM_000077.4(CDKN2A):c.104G>T	NC_000009.11:g.21974723C>A	p.(Gly35Val)	p.(=)
NM_000077.4(CDKN2A):c.104G>A	NC_000009.11:g.21974723C>T	p.(Gly35Glu)	p.(=)
NM_000077.4(CDKN2A):c.103G>A	NC_000009.11:g.21974724C>T	p.(Gly35Arg)	p.(=)
NM_000077.4(CDKN2A):c.101C>T	NC_000009.11:g.21974726G>A	p.(Ala34Val)	p.(=)
NM_000077.4(CDKN2A):c.100G>A	NC_000009.11:g.21974727C>T	p.(Ala34Thr)	p.(=)
NM_000077.4(CDKN2A):c.95T>C	NC_000009.11:g.21974732A>G	p.(Leu32Pro)	p.(=)
NM_000077.4(CDKN2A):c.94C>G	NC_000009.11:g.21974733G>C	p.(Leu32Val)	p.(=)
NM_000077.4(CDKN2A):c.90G>A	NC_000009.11:g.21974737C>T	p.(Ala30=)	p.(=)
NM_000077.4(CDKN2A):c.89C>T	NC_000009.11:g.21974738G>A	p.(Ala30Val)	p.(=)
NM_000077.4(CDKN2A):c.87G>A	NC_000009.11:g.21974740C>T	p.(Arg29=)	p.(=)
NM_000077.4(CDKN2A):c.85C>A	NC_000009.11:g.21974742G>T	p.(Arg29=)	p.(=)
NM_000077.4(CDKN2A):c.85C>T	NC_000009.11:g.21974742G>A	p.(Arg29Trp)	p.(=)
NM_000077.4(CDKN2A):c.82G>A	NC_000009.11:g.21974745C>T	p.(Val28Met)	p.(=)
NM_000077.4(CDKN2A):c.82G>T	NC_000009.11:g.21974745C>A	p.(Val28Leu)	p.(=)
NM_000077.4(CDKN2A):c.81G>C	NC_000009.11:g.21974746C>G	p.(Glu27Asp)	p.(=)
NM_000077.4(CDKN2A):c.81G>A	NC_000009.11:g.21974746C>T	p.(Glu27=)	p.(=)
NM_000077.4(CDKN2A):c.80A>C	NC_000009.11:g.21974747T>G	p.(Glu27Ala)	p.(=)
NM_000077.4(CDKN2A):c.80A>G	NC_000009.11:g.21974747T>C	p.(Glu27Gly)	p.(=)
NM_000077.4(CDKN2A):c.79G>T	NC_000009.11:g.21974748C>A	p.(Glu27*)	p.(=)
NM_000077.4(CDKN2A):c.75A>G	NC_000009.11:g.21974752T>C	p.(Val25=)	p.(=)
NM_000077.4(CDKN2A):c.72G>T	NC_000009.11:g.21974755C>A	p.(Arg24=)	p.(=)
NM_000077.4(CDKN2A):c.71G>T	NC_000009.11:g.21974756C>A	p.(Arg24Leu)	p.(=)
NM_000077.4(CDKN2A):c.71G>A	NC_000009.11:g.21974756C>T	p.(Arg24Gln)	p.(=)
NM_000077.4(CDKN2A):c.71G>C	NC_000009.11:g.21974756C>G	p.(Arg24Pro)	p.(=)
NM_000077.4(CDKN2A):c.69T>G	NC_000009.11:g.21974758A>C	p.(Gly23=)	p.(=)
NM_000077.4(CDKN2A):c.68G>A	NC_000009.11:g.21974759C>T	p.(Gly23Asp)	p.(=)

NM_000077.4(CDKN2A):c.67G>A	NC_000009.11:g.21974760C>T	p.(Gly23Ser)	p.(=)
NM_000077.4(CDKN2A):c.66G>A	NC_000009.11:g.21974761C>T	p.(Arg22=)	p.(=)
NM_000077.4(CDKN2A):c.65G>A	NC_000009.11:g.21974762C>T	p.(Arg22Gln)	p.(=)
NM_000077.4(CDKN2A):c.65G>T	NC_000009.11:g.21974762C>A	p.(Arg22Leu)	p.(=)
NM_000077.4(CDKN2A):c.64C>G	NC_000009.11:g.21974763G>C	p.(Arg22Gly)	p.(=)
NM_058195.3(CDKN2A):c.194-3556C>T	NC_000009.11:g.21974763G>A	p.(Arg22Trp)	p.(=)
NM_000077.4(CDKN2A):c.64C>A	NC_000009.11:g.21974763G>T	p.(Arg22=)	p.(=)
NM_000077.4(CDKN2A):c.62C>T	NC_000009.11:g.21974765G>A	p.(Ala21Val)	p.(=)
NM_000077.4(CDKN2A):c.61G>A	NC_000009.11:g.21974766C>T	p.(Ala21Thr)	p.(=)
NM_000077.4(CDKN2A):c.59C>G	NC_000009.11:g.21974768G>C	p.(Ala20Gly)	p.(=)
NM_000077.4(CDKN2A):c.58G>T	NC_000009.11:g.21974769C>A	p.(Ala20Ser)	p.(=)
NM_000077.4(CDKN2A):c.58G>C	NC_000009.11:g.21974769C>G	p.(Ala20Pro)	p.(=)
NM_000077.4(CDKN2A):c.57C>T	NC_000009.11:g.21974770G>A	p.(Ala19=)	p.(=)
NM_000077.4(CDKN2A):c.54G>T	NC_000009.11:g.21974773C>A	p.(Thr18=)	p.(=)
NM_000077.4(CDKN2A):c.53C>G	NC_000009.11:g.21974774G>C	p.(Thr18Arg)	p.(=)
NM_000077.4(CDKN2A):c.53C>A	NC_000009.11:g.21974774G>T	p.(Thr18Lys)	p.(=)
NM_000077.4(CDKN2A):c.53C>T	NC_000009.11:g.21974774G>A	p.(Thr18Met)	p.(=)
NM_000077.4(CDKN2A):c.52A>C	NC_000009.11:g.21974775T>G	p.(Thr18Pro)	p.(=)
NM_000077.4(CDKN2A):c.51C>T	NC_000009.11:g.21974776G>A	p.(Ala17=)	p.(=)
NM_000077.4(CDKN2A):c.51C>G	NC_000009.11:g.21974776G>C	p.(Ala17=)	p.(=)
NM_000077.4(CDKN2A):c.51C>A	NC_000009.11:g.21974776G>T	p.(Ala17=)	p.(=)
NM_000077.4(CDKN2A):c.50C>T	NC_000009.11:g.21974777G>A	p.(Ala17Val)	p.(=)
NM_000077.4(CDKN2A):c.47T>C	NC_000009.11:g.21974780A>G	p.(Leu16Pro)	p.(=)
NM_000077.4(CDKN2A):c.47T>A	NC_000009.11:g.21974780A>T	p.(Leu16Gln)	p.(=)
NM_000077.4(CDKN2A):c.47T>G	NC_000009.11:g.21974780A>C	p.(Leu16Arg)	p.(=)
NM_000077.4(CDKN2A):c.45G>A	NC_000009.11:g.21974782C>T	p.(Trp15*)	p.(=)
NM_000077.4(CDKN2A):c.45G>T	NC_000009.11:g.21974782C>A	p.(Trp15Cys)	p.(=)
NM_000077.4(CDKN2A):c.44G>A	NC_000009.11:g.21974783C>T	p.(Trp15*)	p.(=)
NM_000077.4(CDKN2A):c.42C>A	NC_000009.11:g.21974785G>T	p.(Asp14Glu)	p.(=)
NM_000077.4(CDKN2A):c.40G>A	NC_000009.11:g.21974787C>T	p.(Asp14Asn)	p.(=)
NM_000077.4(CDKN2A):c.39T>G	NC_000009.11:g.21974788A>C	p.(Ala13=)	p.(=)
NM_000077.4(CDKN2A):c.38C>A	NC_000009.11:g.21974789G>T	p.(Ala13Asp)	p.(=)
NM_000077.4(CDKN2A):c.38C>T	NC_000009.11:g.21974789G>A	p.(Ala13Val)	p.(=)
NM_000077.4(CDKN2A):c.37G>T	NC_000009.11:g.21974790C>A	p.(Ala13Ser)	p.(=)
NM_000077.4(CDKN2A):c.35C>A	NC_000009.11:g.21974792G>T	p.(Ser12*)	p.(=)
NM_000077.4(CDKN2A):c.35C>T	NC_000009.11:g.21974792G>A	p.(Ser12Leu)	p.(=)
NM_000077.4(CDKN2A):c.32C>T	NC_000009.11:g.21974795G>A	p.(Pro11Leu)	p.(=)

NM_000077.4(CDKN2A):c.31C>G	NC_000009.11:g.21974796G>C	p.(Pro11Ala)	p.(=)
NM_000077.4(CDKN2A):c.30G>C	NC_000009.11:g.21974797C>G	p.(Glu10Asp)	p.(=)
NM_000077.4(CDKN2A):c.27G>A	NC_000009.11:g.21974800C>T	p.(Met9Ile)	p.(=)
NM_000077.4(CDKN2A):c.26T>G	NC_000009.11:g.21974801A>C	p.(Met9Arg)	p.(=)
NM_000077.4(CDKN2A):c.26T>C	NC_000009.11:g.21974801A>G	p.(Met9Thr)	p.(=)
NM_000077.4(CDKN2A):c.26T>A	NC_000009.11:g.21974801A>T	p.(Met9Lys)	p.(=)
NM_000077.4(CDKN2A):c.25A>G	NC_000009.11:g.21974802T>C	p.(Met9Val)	p.(=)
NM_000077.4(CDKN2A):c.25A>T	NC_000009.11:g.21974802T>A	p.(Met9Leu)	p.(=)
NM_000077.4(CDKN2A):c.23G>C	NC_000009.11:g.21974804C>G	p.(Ser8Thr)	p.(=)
NM_000077.4(CDKN2A):c.22A>G	NC_000009.11:g.21974805T>C	p.(Ser8Gly)	p.(=)
NM_000077.4(CDKN2A):c.19A>T	NC_000009.11:g.21974808T>A	p.(Ser7Cys)	p.(=)
NM_000077.4(CDKN2A):c.17G>C	NC_000009.11:g.21974810C>G	p.(Gly6Ala)	p.(=)
NM_000077.4(CDKN2A):c.17G>A	NC_000009.11:g.21974810C>T	p.(Gly6Glu)	p.(=)
NM_000077.4(CDKN2A):c.17G>T	NC_000009.11:g.21974810C>A	p.(Gly6Val)	p.(=)
NM_000077.4(CDKN2A):c.16G>A	NC_000009.11:g.21974811C>T	p.(Gly6Arg)	p.(=)
NM_000077.4(CDKN2A):c.15G>T	NC_000009.11:g.21974812C>A	p.(Ala5=)	p.(=)
NM_000077.4(CDKN2A):c.14C>T	NC_000009.11:g.21974813G>A	p.(Ala5Val)	p.(=)
NM_000077.4(CDKN2A):c.14C>A	NC_000009.11:g.21974813G>T	p.(Ala5Glu)	p.(=)
NM_000077.4(CDKN2A):c.13G>A	NC_000009.11:g.21974814C>T	p.(Ala5Thr)	p.(=)
NM_000077.4(CDKN2A):c.11C>G	NC_000009.11:g.21974816G>C	p.(Ala4Gly)	p.(=)
NM_000077.4(CDKN2A):c.9G>A	NC_000009.11:g.21974818C>T	p.(Pro3=)	p.(=)
NM_000077.4(CDKN2A):c.7C>T	NC_000009.11:g.21974820G>A	p.(Pro3Ser)	p.(=)
NM_000077.4(CDKN2A):c.6G>A	NC_000009.11:g.21974821C>T	p.(Glu2=)	p.(=)
NM_000077.4(CDKN2A):c.4G>C	NC_000009.11:g.21974823C>G	p.(Glu2Gln)	p.(=)
NM_000077.4(CDKN2A):c.2T>G	NC_000009.11:g.21974825A>C	p.(Met1?)	p.(=)
NM_000077.4(CDKN2A):c.2T>A	NC_000009.11:g.21974825A>T	p.(Met1?)	p.(=)
NM_000077.4(CDKN2A):c.2T>C	NC_000009.11:g.21974825A>G	p.(Met1?)	p.(=)
NM_000077.4(CDKN2A):c.1A>G	NC_000009.11:g.21974826T>C	p.(Met1?)	p.(=)
NM_000077.4(CDKN2A):c.-2G>A	NC_000009.11:g.21974828C>T	p.(=)	p.(=)
NM_000077.4(CDKN2A):c.-8G>A	NC_000009.11:g.21974834C>T	p.(=)	p.(=)
NM_000077.4(CDKN2A):c.-10G>T	NC_000009.11:g.21974836C>A	p.(=)	p.(=)
NM_000077.4(CDKN2A):c.-10G>A	NC_000009.11:g.21974836C>T	p.(=)	p.(=)
NM_000077.4(CDKN2A):c.-10G>C	NC_000009.11:g.21974836C>G	p.(=)	p.(=)
NM_000077.4(CDKN2A):c.-17G>T	NC_000009.11:g.21974843C>A	p.(=)	p.(=)
NM_058195.3(CDKN2A):c.194-3644C>T	NC_000009.11:g.21974851G>A	p.(=)	p.(=)
NM_000077.4(CDKN2A):c.-30G>A	NC_000009.11:g.21974856C>T	p.(=)	p.(=)
NM_000077.4(CDKN2A):c.-31G>A	NC_000009.11:g.21974857C>T	p.(=)	p.(=)

NM_000077.4(CDKN2A):c.-32G>A	NC_000009.11:g.21974858C>T	p.(=)	p.(=)
NM_000077.4(CDKN2A):c.-33G>C	NC_000009.11:g.21974859C>G	p.(=)	p.(=)
NM_000077.4(CDKN2A):c.-34G>A	NC_000009.11:g.21974860C>T	p.(=)	p.(=)
NM_000077.4(CDKN2A):c.-34G>T	NC_000009.11:g.21974860C>A	p.(=)	p.(=)
NM_000077.4(CDKN2A):c.-40C>A	NC_000009.11:g.21974866G>T	p.(=)	p.(=)
NM_000077.4(CDKN2A):c.-48G>A	NC_000009.11:g.21974874C>T	p.(=)	p.(=)
NM_000077.4(CDKN2A):c.-49C>G	NC_000009.11:g.21974875G>C	p.(=)	p.(=)
NM_000077.4(CDKN2A):c.-49C>A	NC_000009.11:g.21974875G>T	p.(=)	p.(=)
NM_000077.4(CDKN2A):c.-56G>T	NC_000009.11:g.21974882C>A	p.(=)	p.(=)
NM_000077.4(CDKN2A):c.-215C>G	NC_000009.11:g.21975041G>C	p.(=)	p.(=)
